# Using Delaunay triangulation to sample whole‐specimen color from digital images

**DOI:** 10.1002/ece3.7992

**Published:** 2021-08-20

**Authors:** Jennifer J. Valvo, Jose David Aponte, Mitch J. Daniel, Kenna Dwinell, Helen Rodd, David Houle, Kimberly A. Hughes

**Affiliations:** ^1^ Department of Biological Science Florida State University Tallahassee Florida USA; ^2^ Department of Cell Biology and Anatomy University of Calgary Calgary AB Canada; ^3^ Department of Ecology and Evolutionary Biology University of Toronto Toronto ON Canada

**Keywords:** color pattern analysis, color quantification, color variation, guppy color pattern, image analysis

## Abstract

1. Color variation is one of the most obvious examples of variation in nature, but biologically meaningful quantification and interpretation of variation in color and complex patterns are challenging. Many current methods for assessing variation in color patterns classify color patterns using categorical measures and provide aggregate measures that ignore spatial pattern, or both, losing potentially important aspects of color pattern.
2. Here, we present *Colormesh*, a novel method for analyzing complex color patterns that offers unique capabilities. Our approach is based on unsupervised color quantification combined with geometric morphometrics to identify regions of putative spatial homology across samples, from histology sections to whole organisms. *Colormesh* quantifies color at individual sampling points across the whole sample.
3. We demonstrate the utility of *Colormesh* using digital images of Trinidadian guppies (*Poecilia reticulata*), for which the evolution of color has been frequently studied. Guppies have repeatedly evolved in response to ecological differences between up‐ and downstream locations in Trinidadian rivers, resulting in extensive parallel evolution of many phenotypes. Previous studies have, for example, compared the area and quantity of discrete color (e.g., area of orange, number of black spots) between these up‐ and downstream locations neglecting spatial placement of these areas. Using the *Colormesh* pipeline, we show that patterns of whole‐animal color variation do not match expectations suggested by previous work.
4. *Colormesh* can be deployed to address a much wider range of questions about color pattern variation than previous approaches. Colormesh is thus especially suited for analyses that seek to identify the biologically important aspects of color pattern when there are multiple competing hypotheses or even no a priori hypotheses at all.

## Graphical abstract

There is considerable interest in quantifying color patterns in order to investigate the role that color plays in ecological and evolutionary processes, including competition, mate choice, kin selection, predation, and speciation. The Colormesh approach is a novel, unsupervised method for sampling color from digital images, enabling highly multivariate quantification of color patterns. We demonstrate the utility of the Colormesh approach by evaluating patterns of color variation in the Trinidadian guppy, a model system for studies of the role of color in local adaptation and in the maintenance of genetic diversity.

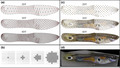

## 1 INTRODUCTION

Measurement of color is used in many fields of study including, for example, astronomy (Bessell, 2005; Robinson et al., 2010), medicine (Bhargava & Madabhushi, 2016; Limkin et al., 2017), and agriculture and food science (Dell'Aquila, 2009; Pathare et al., 2013). In biology, ecological, evolutionary, and mechanistic investigations of organismal color have been prominent in the scientific literature since the 19th century and have provided key insights into many aspects of organismal function and evolution, such as the role of coloration in thermoregulation, crypsis, aposematism, mimicry, mate choice, and speciation (Cott, 1940; Cuthill, 2019; Darwin, 1859; Jennions & Petrie, 1997; Mallet & Joron, 1999; Map pes et al., 2005; Mclean & Stuart‐Fox, 2014). In recent years, color analyses have expanded to include more intricate investigations of how both color and patterning are produced (e.g., Manukyan et al., 2017; Shawkey & D'Alba, 2017) and perceived (e.g., Kelber, 2016; Stoddard et al., 2014), allowing for novel questions to be asked about the role of organismal coloration.

Despite the importance of coloration, its complexity and patterning can pose serious challenges to measurement and interpretation. Many different approaches that often rely on human perception of color have been used to quantify color patterns and how they vary among organisms, populations, and species. Early methods of assessing color in biology relied on categorical schemes, such as assigning patterns to discrete morphs (Brown & Clegg, 1984; Semler, 1971; Tan & Li, 1934). Using photographs, later studies quantified color with measures of total area or percent coverage of discrete categories of color (e.g., “orange,” “blue”), as well as the number of particular color pattern elements (e.g., numbers of “spots” of particular colors) (Houde, 1987; Olsson, 1994; Petrie & Halliday, 1994). Although subjective, these relatively low‐dimensional measures are useful in addressing biological questions such as: Is there covariation between female preference and ornamental traits (Ellers & Boggs, 2003; Endler & Houde, 1995)? Are some color morphs more successful at attracting mates or surviving (Borer et al., 2010; Petrie, 1992; Sinervo & Lively, 1996)? Do morphs differ in life‐history traits (Emaresi et al., 2014; Svensson et al., 2001)? Does morph frequency vary with habitat type (Ahnesjö & Forsman, 2006; Power et al., 2005)?

In many studies, human perception was used to determine color categories and the boundaries of color pattern elements in order to quantify color patterns. Recent technological advancements in digital imaging and computation have enabled more objective characterization of color. For example, individual pixels in digital images can be assigned values in a color space (e.g., red, green, and blue color channels in RGB color space), and spectrophotometry can capture the entire reflectance spectra of specific locations on an organism. In digital image analysis, every pixel can be described by a quantitative value for each color channel. Pixels can then be grouped into discrete color categories using clustering or thresholding to perform image segmentation. Applications such as *ImageJ* (Schneider et al., 2012), *patternize* (Van Belleghem et al., 2018), and *colordistance* (Weller & Westneat, 2019) offer different methods for grouping pixels of similar color to assess overall patterns (i.e., perform image segmentation). These methods allow the user to set range limits for RGB values, which then enables automatic color segmentation. Alternatively, a user can define the number of color categories in which to bin pixels and then use *k*‐means clustering to identify pixel groups. The *patternize* package provides an additional segmentation option known as watershed transformation. In watershedding, the image is treated like a topographic map where a pixel within a user‐defined region is selected and surrounding pixels are binned with the focal pixel if RGB values fall within a specified threshold; when pixels fall outside of the threshold, this becomes a border to that color pattern element and a new element is initiated; this process continues until the image is entirely segmented.

Image segmentation approaches have been useful in addressing many questions. For example, *ImageJ* has been used for counting pixels within a specified color range to identify diseased tissue (Hadi et al., 2011; Papadopulos et al., 2007; Schindelin et al., 2015), *patternize* was used to compare the similarity of distributions of three discrete color categories among species of reef fish (Hemingson et al., 2019), and *colordistance* was used to visualize badge color differences between two lizard populations (Orton et al., 2019). In certain applications, the use of digital photography, along with computer software to group pixels into categories of color, has decreased subjectivity of color classification and increased the accuracy and reproducibility of color data collection (e.g., Laurinaviciene et al., 2011; Rizzardi et al., 2016). However, because consumer cameras are designed to capture color in a manner that mimics human perception, it is important to understand the limitation imposed by the human perception of color (Troscianko & Stevens, 2015).

Methods other than segmentation of digital images are necessary if information on whole color pattern variation (simultaneous evaluation of chromatic and spatial variation) is required. The *patternize* package uses presence or absence of a color at a location to assess spatial variation of color categories that are defined in the segmentation process. This limits the use of *patternize* to assessment of variation in the spatial distribution of one category of color at a time. Therefore, *patternize* works well for assessing pattern elements having clear boundaries and when spatial patterns of discrete colors vary, but the color itself does not change. Following color segmentation, *colordistance* calculates the distance between the color distributions of two images within a color space (either RGB or CIELAB). While *colordistance* accounts for how much of each color is present in an image and how close those colors are in color space, the location of these colors within the pattern is lost. In order to evaluate whole color pattern variation, measurement of spatially explicit variation in color is needed, and tools to evaluate color patterns in this manner have been lacking.

Although clustering of pixels can be automated, pixel values used to determine boundaries between color categories are typically set by the user, which introduces a potentially problematic level of human subjectivity (Davidoff & Fagot, 2010; Siuda‐Krzywicka et al., 2019; but see Bergeron & Fuller, 2018), as well as information loss due to discretizing continuous color variation. Reflectance spectrometry is an objective measure of color that quantifies the wavelengths and intensities of light reflected from a small point sample over a continuous range of wavelengths (Andersson et al., 1998; Endler, 1990; Gomez & Théry, 2007; Zuk & Decruyenaere, 1994). Heterogeneous patterns can be sampled by collecting data from multiple sampling points in a standardized manner (Cuthill et al., 1999; Endler, 2012; Endler & Mielke, 2005). This technique has been used to acquire objective color data in many ecological and evolutionary studies, often in combination with models of the visual sensitivity of receivers of the color information, for example, potential predators, mates, or pollinators (Cortesi & Cheney, 2010; Dyer et al., 2012; Isaac & Gregory, 2013; Stoddard & Stevens, 2011). Understanding how a receiver perceives a visual signal is clearly important for studies of behavioral and ecological interactions based on color. The R package, *pavo2*, provides a framework for processing and combing spectral and spatial data with visual models (Maia et al., 2019). Because this package incorporates analyses such as between‐pattern contrasts (Endler & Mielke, 2005), adjacency analysis (Endler, 2012), and boundary strength analysis (Endler et al., 2018) to model perception of color patterns, a large number of spectrophotometric sample points are required when color patterns are complex. However, for organisms with complex color patterns, the level of sampling required to compare color patterns among a large number of individuals is not currently feasible using reflectance spectrometry.

Here, we present a new approach for sampling color patterns from digital images using *Colormesh*, a package within the R statistical computing environment (R Core Team, 2019), that is spatially explicit, high dimensional, high throughput, and does not rely on subjective determination of the number or type of color categories. *Colormesh* is an unsupervised approach that measures multidimensional color data by dense sampling of quantitative color values across the entire sample area and therefore does not require clearly defined color pattern elements. To accomplish this, our method uses Delaunay triangulation to identify homologous sampling points on images that were standardized to a consensus shape with geometric morphometrics software. With *Colormesh*, we (a) enable analysis of color patterns that are highly variable, spatially complex, and/or lack well‐organized color pattern elements (e.g., spots or stripes), (b) capture the continuous, high‐dimensional nature of color variation, (c) use an unsupervised method to determine points on standard digital images from which to sample color values, and (d) allow for flexibility in color sampling density and the size of the area sampled.

We demonstrate the utility of *Colormesh* using digital images of Trinidadian guppies (*Poecilia reticulata*), in which the evolution of color is a topic of active study. We photographed male guppies from eight natural and three experimental populations (described below). Complex color patterns in this species are male limited, highly heritable, and highly variable both within and between populations (Brooks & Endler, 2001b; Endler, 1995; Gordon et al., 2015; Houde, 1997; Hughes et al., 2005; Kemp et al., 2008; Magurran, 2005; Winge, 1927). Male guppy coloration has become a model system for studies of local adaptation (Gordon et al., 2015; Houde, 1997; Millar et al., 2006) and for the maintenance of ecologically important variation (Brooks & Endler, 2001a; Evans et al., 2008; Hughes et al., 1999, 2013; Olendorf et al., 2006; Valvo et al., 2019). Guppies have repeatedly evolved in response to different ecological conditions above (upstream) and below (downstream) barrier waterfalls in Trinidadian rivers and streams (Endler, 1978; Magurran, 2005), and upstream populations are known to be descendants of downstream populations within rivers (Alexander et al., 2006; Becher & Magurran, 2000; Crispo et al., 2006; Magurran et al., 1992; Shaw et al., 1992; Suk & Neff, 2009; Willing et al., 2010). Downstream habitats are often referred to as high predation due to the presence of one or more species of large piscivorous fish (Endler, 1980; Reznick et al., 1996). In addition, many downstream sites have relatively open forest canopy and high primary productivity (Grether et al., 2001). In contrast, upstream habitats, typically referred to as low predation, generally contain one main, smaller guppy predator, *Anablepsoides hartii* (formerly, *Rivulus hartii*), that preys on juveniles and small adult guppies (Gilliam et al., 1993; Reznick et al., 1996); these low‐predation habitats typically have a relatively closed canopy and low primary productivity (Grether et al., 2001; Reznick et al., 2001). This repeated ecological transition has led to extensive parallel evolution of many phenotypes (Reznick & Endler, 1982; Reznick & Bryga, 1987, 1996; Reznick, 1989; Reznick, Rodd, et al., 1996; Torres Dowdall et al., 2012; reviewed in Houde, 1997; Magurran, 2005), including male color (Endler, 1978, 1983, 1991).

It has been proposed that these ecological differences between down‐ and upstream sites, including predation intensity, also select for differing levels of color polymorphism between populations, through processes that generate negative frequency‐dependent selection (NFDS) (Endler, 1980; Fraser et al., 2013; Olendorf et al., 2006). In natural populations, males bearing rare color patterns have higher survival than males bearing common patterns, and this effect is stronger in low‐predation sites than in high‐predation ones (Olendorf et al., 2006). *Anablepsoides* *hartii*, which impose a greater predation risk in upstream sites, learn to be a more effective guppy predator the more it is exposed to a particular male color pattern (Fraser et al., 2013). Female guppies also prefer males bearing rare or unfamiliar color patterns (Eakley & Houde, 2004; Graber et al., 2015; Hampton et al., 2009; Hughes et al., 2013; Mariette et al., 2010; Zajitschek & Brooks, 2008; Zajitschek et al., 2006), and this preference has been documented in both high‐ and low‐predation sites (Valvo et al., 2019). Taken together, these results suggest that low‐predation sites should exhibit more variation in male color patterns, since NFDS by *A. hartii* should be stronger, and sexual selection by female preference is equally strong in the low‐ and high‐predation sites. To test this prediction, whole color pattern must be quantified, which to our knowledge has not previously been attempted.

Here we use *Colormesh* to address three questions about color variation and evolution in Trinidadian guppies. We first asked if our method could successfully classify individual fish by their population of origin. We then asked if the direction of color evolution between up‐ and downstream populations was consistent across river drainages. Finally, we asked if the within‐population variance in color differed consistently between up‐ and downstream populations and evaluated the contribution of color at different locations on the fish to within‐population variation in color.

## 2 METHODS

### 2.1 Overview

Each section below provides instructions for the preparation, and sampling of 2D digital images with the *Colormesh* package. To sample color from homologous locations among images, subjects must first be processed to a standardized size and shape. Image processing (landmark placement and image transformation to a consensus shape) within the *Colormesh* package is described in brief below. Alternatively, image processing may be completed externally using other geometric morphometric software (e.g., the R package, *Morpho* (Schlager, 2017), the *TPS series* software (Rohlf, 2015)) and then imported and sampled using the *Colormesh* sampling pipeline. Following image processing, we then describe the *Colormesh* sampling pipeline that uses Delaunay triangulation as an unsupervised method to determine points from which to sample color. Finally, we present how we used *Colormesh* to address evolutionary questions relevant to the guppy study system using data extracted with the *Colormesh* package.

*Colormesh* can be downloaded for free at https://github.com/J0vid/Colormesh. The GitHub site provides instructions for download and a forum for posting questions or issues. Explanatory details and example code for using *Colormesh* to process images (or import data obtained through external processing) and color sampling are provided on the Readme page of the GitHub site, as well as in a tutorial provided in the Appendix S1.

### 2.2 Required input for color sampling

*Colormesh* requires two CSV files to provide information during processing: one having the unique specimen image names and associated identification information and the other to provide the known RGB values of the colors on the standard for the calibration process. To sample RGB color values from pixels, *Colormesh* requires two sets of images as input: one set of images that have been processed using landmark‐based geometric morphometric methods (either within the *Colormesh* package or externally) for color sampling and the original set of images containing a color standard for the image‐specific color calibration. Lastly, two arrays containing landmark coordinate data are required: one of the consensus shape and another having the locations to sample the color standard. Landmark placement and generation of consensus shape images within *Colormesh* (described below) will produce the required coordinate data arrays; externally generated landmark coordinate data are easily imported (examples available in the Appendix S1 and on the Github site).

#### 2.2.1 Image processing (landmarking and image transformation)

To generate the required image inputs, digital photography is used to capture 2D images of specimens that also include a size scale and color standard. Several common image formats (e.g., JPEG, PNG, BMP, and TIF), as well as the raw image formats unique to Canon, Nikon, and Olympus brand cameras (CR2, NEF, and ORF, respectively) are compatible with *Colormesh*; the *magick* package (Ooms, 2021) is used to read images because of its support for a large variety of image formats. Digital images that are to be compared must all have the same pixel dimensions (length × width), and image names must be unique and match the image names in the CSV file containing the image information. All digital image files must be saved in the same folder.

The first step in transforming specimen images to a consensus shape is the placement of landmarks on each specimen. To identify the scale, location of the color standard, and place landmarks, the user invokes the *landmark.images* function within *Colormesh*. This presents each image to the user and utilizes the user‐friendly digitization capabilities provided by the R package *geomorph* (Adams et al., 2020) to generate the array containing the landmark coordinates associated with each specimen image. Following scale setting, traditional landmarks are first placed at several specific locations on the specimen that can be consistently identified across samples (e.g., fin attachment sites in our guppy case study). Semilandmarks can also be placed to represent a curve or surface on the specimen where locations that are biologically homologous are not easily identifiable (Bookstein, 1997). The quantity and placement of semilandmarks are up to the user and should be varied depending on the complexity and level of variation of the specimen's shape (Gunz & Mitteroecker, 2013; Watanabe, 2018). To infer a smooth curve between traditional landmarks when generating the consensus shape, semilandmarks are slid into place before analysis (Gunz & Mitteroecker, 2013).

For the color calibration process, the *landmark.images* function is invoked a second time. This array is generated to provide landmark coordinates identifying where each image is to be sampled for calibration (described below); for this array, a scale is not defined since color calibration does not use shape analysis.

The last step of image processing is the generation of consensus shape images using landmark‐based image transformation. The user invokes the *tps.unwarp* function which employs the utilities of two R packages: *geomorph* (Adams & Otárola‐Castillo, 2013) and *imager* (Barthelme, 2020) to calculate the consensus specimen shape and produce the transformed images, respectively. First, a Generalized Procrustes Analysis (GPA) is performed using the landmark coordinate data from each specimen image to compute the consensus specimen shape. If semilandmarks are defined, by default they slide to minimize bending energy between individuals specimens and the consensus shape (Gunz & Mitteroecker, 2013); otherwise, all landmark coordinates are treated equally in the GPA that estimates the consensus shape. The function generates an array that contains the landmark coordinates of the computed consensus shape. This consensus shape is then used as the target for transforming each specimen image to the consensus shape using a thin plate spline (TPS) image transformation. The resulting transformed images are then written to a directory, specified by the user, as lossless, compressed, PNG format images.

#### 2.2.2 Color sampling pipeline

The *Colormesh* sampling pipeline is based on unsupervised color quantification at individual sampling points across the transformed photograph of the specimen. Novel to *Colormesh* is the use of Delaunay triangulation, which generates a surface of triangles from a finite set of points using three nearest points whose circumcircles do not contain any other points in the set. In computer graphics, Delaunay triangulation is used to represent a large number of points within a boundary surface (i.e., a finite set of points) with a reduced number of points that function as a concise representation of the shape (De Berg et al., 2008; Bala & Sekhon, 2011, see Aurenhammer, 1991 for use of Delaunay triangulation in geometric data structure); Delaunay triangulation has previously been used to identify nonlandmarked points used for shape analysis (Márquez et al., 2012). With *Colormesh*, the centroids of the triangles serve as the sampling template in that their coordinates identify comparable pixels on each transformed specimen image that will be sampled for RGB values.

The first step in the color sampling pipeline is the generation of the sampling template. The user invokes the *tri.surf* function within *Colormesh*, which uses two auxiliary R packages (*sp* (Pebesma & Bivand, 2005) and *tripack* (Renka et al., 2016)) to calculate the surface of sampling points from the landmark coordinate data of the consensus shape array. The granularity, or density, of sampling is user‐controlled by the number of rounds of triangulation that is defined in the function; additional rounds of triangulation beyond the first use the centroid coordinates from the previous round of triangulation as the vertices for the subsequent round. Therefore, each additional round of triangulation increases the density of sampling points (e.g., Figure 2a) and also increases downstream processing times. The output of the *tri.surf* function is the sampling template containing the coordinates of the pixel located at the centroid of each triangle.

The next step in the color sampling pipeline is sampling RGB values from a sampling circle that is centered on each pixel identified in the sampling template. To sample RGB color values, the user invokes the *rgb.measure* function. The size of the sampling circle (e.g., Figure 2b) is defined in this function allowing the user to control the level of pixel averaging for color sampled at a location; in contrast to the density of sampling points, the sampling circle size does not influence downstream processing time. This function uses the *imager* package (Barthelme, 2020) to extract R, G, and B values from pixels included in the sampling circle and calculates the mean for each color channel. The final output of this function generates a list of extracted values for each color channel for each image and provides the sampling template coordinates. For visualization, *Colormesh* provides several options for plotting the color values extracted from the consensus shape images (e.g., Figure 2c, d).

The *Colormesh* approach is flexible in that it allows user‐controlled sampling density and circle sizes. However, to reduce the level of subjectivity, the extracted data may be used to inform these decisions. We demonstrate a method using our sample data to determine which sampling scheme (sampling density and circle size) best differentiates among specimens in the *Multivariate classification and differentiation among populations* section below.

#### 2.2.3 Color calibration

Providing the known RGB values of the color standard enables *Colormesh* to correct the sampled color values. Calibration is performed by the *rgb.calibrate* function which calculates an image‐specific color channel correction vector. First, *Colormesh* reads in the array of coordinate data from landmarks placed on the color standard in the images. The RGB values are then sampled from the pixels contained within a sampling circle centered on the coordinates provided by the array. The mean deviation from the known color channel values of the standard included in each image is calculated and the channel‐specific correction is applied to the measured values for that image.

### 2.3 *Colormesh* applied to the Trinidadian guppy system

#### 2.3.1 Populations sampled

To compare color patterns in a diverse set of populations, we photographed wild male guppies captured during the dry season in May of 2016 and 2017. These fish were collected from six rivers belonging to three major drainage systems in the Northern Range in Trinidad (see Table S1 for GPS locations): the Caroni drainage (Aripo, El Cedro, and Guanapo Rivers), Northern drainage (Marianne and Paria Rivers), and the Oropuche drainage (Turure River). In the Aripo, El Cedro, Guanapo, Marianne, and Turure Rivers, we sampled males from up‐ and downstream habitats that are characterized by important ecological differences (Endler, 1995; Houde, 1997; Magurran, 2005). We also sampled one additional population, the Houde Tributary of the Paria River, which is a low‐predation site and does not have an associated high‐predation site (Houde, 1997; Magurran, 2005). Of the 11 populations sampled, eight were naturally occurring populations and three were experimental populations. The experimental populations were the high‐ and low‐predation populations sampled from the Turure river, where guppies were introduced in 1957 (Becher & Magurran, 2000) and the low‐predation El Cedro population where guppies were introduced in 1987 (Reznick & Bryga, 1987).

For logistical reasons, males from the Aripo, El Cedro, and Paria Rivers, and the low‐predation population of the Marianne River were collected and photographed in 2016. Males from the Guanapo, Turure, and the high‐predation population of the Marianne River were collected and photographed in 2017. See Appendix Methods for details on fish collection and digital photography. We photographed a total of 485 male fish, 24–57 per population (See Table S1 for sample sizes).

#### 2.3.2 Image processing and color sampling

**FIGURE 1:**
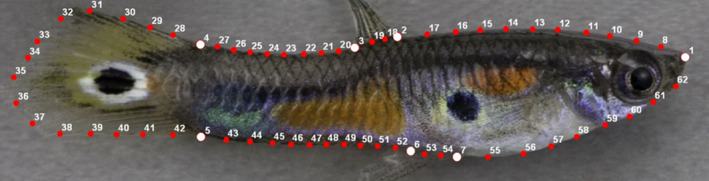
An example of an image opened within the *tpsDig2* software that was used to place landmarks on an image. A total of 62 landmarks are placed in the same order on every image. Seven traditional landmarks (white circles) are placed first in locations that can be consistently identified on each fish. Between the traditional landmarks, 55 semilandmarks (red circles) are placed; the number of semilandmarks between each traditional landmark was consistent between images and the user placed them approximately equally spaced apart between the two traditional landmarks

Image processing was performed externally using the *TPS Series* morphometrics software (Rohlf, 2015); details of image processing specific to the *TPS series* software are included in the Appendix: Methods. In brief, digital images used in the guppy example analyses were taken in the camera's raw image format (.cr2) and converted to the .TIF format with an output color space of sRGB in Photoshop CC 2018. Each digital photograph of a male guppy included a size and color standard. After setting the image scale (pixels/cm), a total of 62 landmarks were placed around the perimeter of each fish. Seven traditional landmarks were first placed at the following locations: the tip of the snout, anterior and posterior connection points of the dorsal fin to the body, dorsal and ventral connection points of the caudal fin to the caudal peduncle, posterior and anterior connection points of the gonopodium to the body (Figure 1). Fifty‐five semilandmarks were placed along the edge of the fish, among the traditional landmarks, in a counterclockwise direction (Figure 1). For the color calibration process, a landmark was placed on each of the five colors in the color standard included in each photograph. Landmark placement on all photographs was performed by K.D.

Populations were initially processed separately to generate population‐specific consensus shapes. To avoid biasing fish shape toward a particular population since the numbers of fish sampled for populations varied, an overall‐consensus shape was then generated using the 11 population‐specific consensus shapes as inputs. Individual images were then transformed to the overall‐consensus shape and saved as TIF image files, thus providing the consensus‐shaped image set required for the *Colormesh* sampling pipeline. Following external image processing, all required files were read into *Colormesh* and we proceeded with the color sampling pipeline.

Color data were then collected from the processed images using 12 different sampling schemes (sampling densities of two, three, and four Delaunay triangulations, each with sampling circle diameters of one, three, five, and nine pixels). Color data were extracted from guppy images using the auxiliary package *EBImage* (Pau et al., 2010), but for future users, *EBImage* has now been replaced by the *imager* package in the current version of *Colormesh*. Because the known RGB values of the five colors included on the color standard were given on a scale of 0–255, values were divided by 255 to match the scale of values extracted which range from 0 to 1 (Table S2).

#### 2.3.3 Multivariate classification and differentiation among populations

We used Discriminant Analysis of Principal Components (DAPC) in the R package, *adegenet* (Jombart, 2008) to determine if color pattern was associated with population sampling site. DAPC can also be used in a cross‐validation framework to determine if data can be used to classify observations into predefined categories. We used DAPC to determine which of the sampling schemes and pixel sample circle sizes (described above) allowed us to best discriminate among populations. Because DAPC requires the user to specify how many PCs to retain, we first used the *xvalDapc* function in the R package *adegenet* (Jombart, 2008) to assess the proportion of successful assignments and root mean squared error (RMSE) for a varying number of retained PCs. We specified that 80% of each sample be used for training and 20% for validation. Cross‐validations were performed at nine different retention levels of PCs in increments of 50 ranging from 50 to 450. We set the number of replicates to be carried out at each PC retention (*n.rep*) = 100. We compared the lowest RMSE and proportion of successful placements for each of the 12 sampling schemes to determine which sampling scheme would be used for the remaining analyses.

#### 2.3.4 Direction of evolution within and between rivers

We used the guppy *Colormesh* data to compare the direction of evolutionary divergence between populations with known ancestor‐descendant relationships. This analysis tests the hypothesis that color pattern evolution has evolved convergently each time high‐predation guppies have moved upstream to invade low‐predation habitats. For four of the six rivers that we sampled (Aripo, El Cedro, Guanapo, and Turure), the paired high‐ and low‐predation populations were sampled in the same year. This meant the images had the same pixel resolution prior to the image processing described above, so the high‐ and low‐predation populations within these four rivers could be compared directly. For this analysis, each fish had R, G, and B values measured at 2,462 positions for a color vector of length = 7,386 variables.

To produce an average color pattern for a given population, we calculated the mean value of each color channel at each sampling point. Thus, each population had a mean color vector consisting of 2,462 values for each color channel. Because low‐predation populations are known to be descendants of high‐predation populations within each river, the difference between the population mean color vectors measures the direction of color evolution within a river. If color pattern evolution was similar among rivers, these difference vectors should be more similar than that expected by chance. We measured the similarity of these vectors by calculating the angle between the vectors of each pair of rivers. If colors evolved similarly in two river drainages, the vectors indicating direction of evolution will have a smaller angle between them than random vectors. The angle between the vectors can also be expressed as a vector correlation (*r*), which is mathematically equivalent to Pearson's correlation coefficient.

In order to calculate a confidence interval for this estimate of the correlation in direction of color evolution between each pairing of the four rivers, we first bootstrapped whole‐fish color patterns. Populations were resampled 1,000 times using the *boot* function in the R *boot* package (Canty & Ripley, 2019; Davison & Hinkley, 1997). Whole fish color patterns were bootstrapped in order to retain the associations of RGB values within and among the sampling points. For each bootstrap sample, we calculated the population mean color pattern as described above. This produced 1,000 bootstrap estimates of the population mean color pattern. To generate 1,000 estimates of the direction of evolution within a river, we subtracted the mean vector of the first bootstrap sample in the high‐predation population from the mean vector of the first bootstrap sample in the low‐predation population; this was repeated for the remaining 999 bootstrap samples from the high‐ and low‐predation populations. We then calculated the vector correlation between each of the 1,000 bootstrap estimates of color evolution within the rivers. Pairwise comparisons of four rivers generated six distributions of correlation coefficients.

To determine whether the correlation between pairs of rivers was more similar than expected by chance, we calculated a null expectation for the correlation between random vectors. We first generated vectors to simulate two rivers where the expected direction of evolution within each river was random. This was done using the *mvrnorm* function in the *MASS* package in R (Venables & Ripley, 2002). We created two matrices, each with 1,000 random vectors of dimension = 7,386, mu = 0 (vector giving the mean of zero for each variable of the matrix), and Sigma defined as the identity matrix (ones on the diagonal and zeros on the off‐diagonal) to simulate random direction of evolution estimates. Similar to our bootstrap samples, we then paired each vector between the two simulated river estimates and calculated the mean correlation between random vectors. If the estimate of the vector correlations for each river comparison was above or below the mean correlation between random vectors, we determined the correlation between rivers in the direction of color evolution was more correlated than expected by chance.

#### 2.3.5 Phenotypic variance in color between predation regimes

We compared phenotypic variation in male coloration between high‐ and low‐predation populations using the same data set from the rivers included in our analysis of the direction of color evolution (Aripo, El Cedro, Guanapo, and Turure). We calculated the trace of the variance‐covariance matrix to determine the phenotypic variance in color for each real population. Our test statistic was the trace of the high‐predation population minus the trace of the low‐predation population.

We used permutation to test if the phenotypic variation differed between high‐ and low‐predation populations within a river. We permuted the label of high predation or low predation for each whole‐fish color pattern within a single river using the *sample* function in base R (V3.5.3), created 1,000 permutation samples, and calculated the difference between traces. The distribution of these 1,000 measures of difference in variance was used to represent the null distribution.

We were also interested in characterizing the whole‐specimen spatial pattern of within‐population variance in color. To do so, we compared the within‐ and between‐population components of variance for each color channel at every *x*, *y* coordinate in the data set. We estimated variance components with restricted maximum‐likelihood using *Proc Varcomp* of SAS version 9.4 (SAS Institute, Cary, NC), with the color value (R, G, or B) at a given *x*, *y* position as the dependent variable, year as a fixed effect, and population ID as a random effect.

## 3 RESULTS

### 3.1 Multivariate classification and differentiation among populations

**FIGURE 2:**
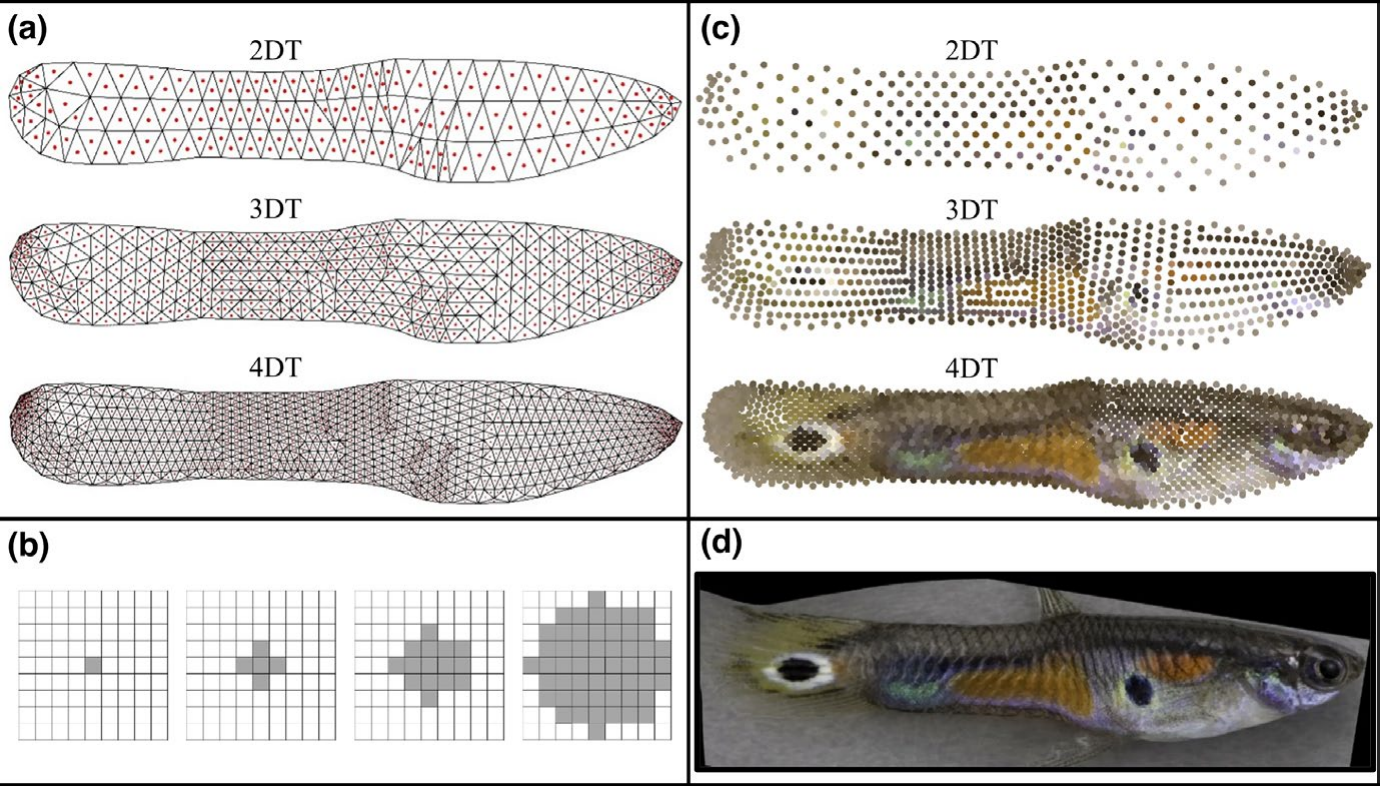
Panel (a) shows the mesh generated from the user‐defined sampling scheme of 2, 3, and 4 rounds of Delaunay triangulation (DT). The triangle mesh is used to determine sampling location (red dot at each triangle centroid). The triangle centroids defined by the previous round of triangulation were used to generate the subsequent round of triangulation. Panel (b) shows the pixels sampled depending on the user‐defined sampling circle radius size. Shown are the sampling circles with diameter = 1, 3, 5, and 9 pixels, from left to right. Panel (c) shows a plot of the RGB values sampled from an unwarped image (Panel d) at different sampling densities (DTs). Here we only show the sampling design where the sampling circle diameter = 1 (only the triangle centroid RGB values were measured). To visualize the color sampled at single pixels, the sampled RGB values from each pixel are reproduced as larger circles

We first sought to determine a combination of sampling density and sampling circle size that would enable us to classify the 11 guppy populations with the highest precision. As the number of rounds of Delaunay triangulations (DT) increased, the root mean squared error (RMSE), averaged over all sampling circle sizes, consistently decreased (RMSE averaged across years: 2DT = 0.18, 3DT = 0.15, 4DT = 0.13; Table S3), and the mean successful assignment of the validation set increased slightly (proportion of successful assignment averaged across years: 2DT = 0.87, 3DT = 0.90, 4DT = 0.91; Table S3). To visualize the difference in sample density, we reproduced an example image by plotting the sampled RGB values at each of the three sampling densities (Figure 2c); clearly, the increased sampling density improves the representation of the complex guppy color pattern to human perception.

To choose the sampling circle diameter for our analyses, we again evaluated the cross‐validation results (Table S3). At the highest sampling density (4DT), classification success was high and nearly constant irrespective of sampling circle diameter (range: 0.91–0.93). When images were reproduced using color sampled at the four different sampling circle diameters (1, 3, 5, and 9 pixels), there was no noticeable difference in image quality that would suggest that one of the four sampling circle diameter sizes would best represent an image (Figure S1). We therefore decided to use sampling circle diameter of 1 (1 pixel sampled, no pixel averaging) because it produced the lowest RMSE and highest success rate in both years, when years were analyzed separately (Table S3). This sampling scheme produced a color vector of length 7,386 (2,462 sampling points by three color channels).

**FIGURE 3:**
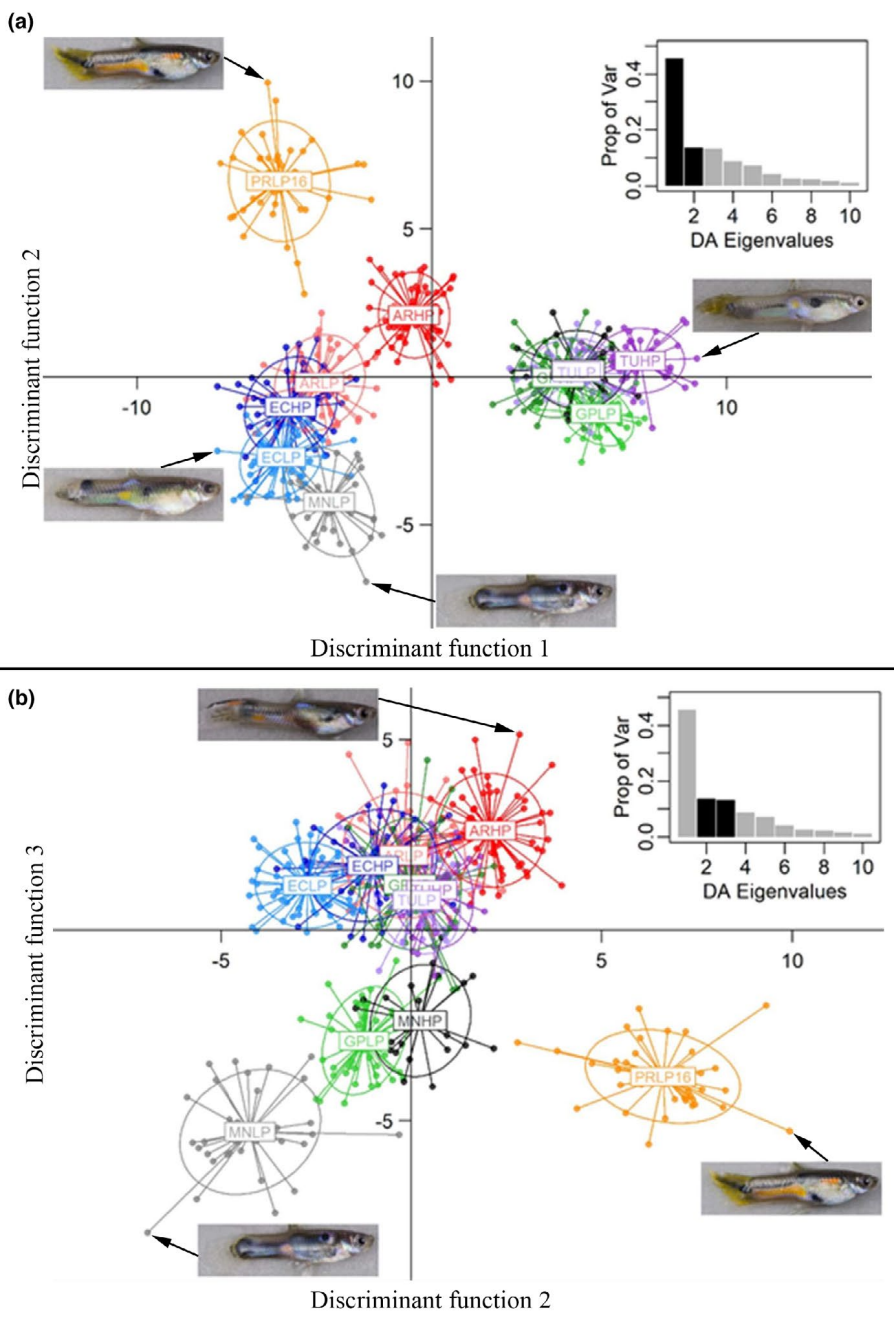
Scatter plot from the discriminant analysis of principal components (DAPC) where population membership was predefined (11 populations). Points represent the coordinates of individuals, and populations are within inertial ellipses. The four letters at the center of the inertial ellipses represent the population: The first two letters identify the river (AR = Aripo, EC = El Cedro, GP = Guanapo, MN = Marianne, PR = Paria, TU = Turure), followed by two letters that identify the predation regime (HP = high predation, LP = low predation). High‐ and low‐predation populations sampled within the same river are represented by dark and light versions of the same color, respectively. The barplot (inset) shows the proportion of variation explained by the discriminant analysis eigenvalues; dark bars correspond to the axes presented in the scatter plot of each panel section. Discriminant function axes one and two are plotted in panel (a), and axes two and three are plotted in panel (b). Original images (prior to unwarping) of individuals located at the extremes of each axis are shown for each panel. The color data used for the DAPC were collected from photographs unwarped to a consensus shape, and red, green, and blue (RGB) color channels were sampled from 2,462 pixels

Using this sampling scheme, DAPC analysis generated discriminant functions representing principal components that best differentiated guppy populations. Figure 3 shows the positions of all fish in the 11 populations on the first three discriminant function axes. Discriminant functions one, two, and three accounted for 45.6%, 13.6%, and 13.2% of the variation in color patterns, respectively. Axis one separated populations predominantly by the year in which they were sampled; populations collected in 2016 were clustered to the left and did not overlap with the samples collected in 2017. Images from 2017 were photographed under slightly different conditions than in 2016 and were clustered to the right in the scatterplot (Figure 3a). In contrast, axis two consistently separated high‐ and low‐predation populations of the same river, with high‐predation populations higher (more positive) on axis two than the corresponding low‐predation population (Figure 3a). Indeed, even though the two Marianne populations were sampled in different years and there were slight differences in the resolution of the images, the direction of separation along axis two followed this pattern. Axis three also consistently separated high‐ and low‐predation population pairs, with high‐predation populations always placed higher (more positive) on axis three than their low‐predation counterparts (Figure 3b). In Figure 3b, which plots axes two and three, it is evident that these two axes together consistently differentiate low‐ and high‐predation population pairs, with low‐predation populations always below and to the left (more negative on both axes) of their high‐predation counterpart.

### 3.2 Comparison of direction of evolution within and between rivers

**FIGURE 4:**
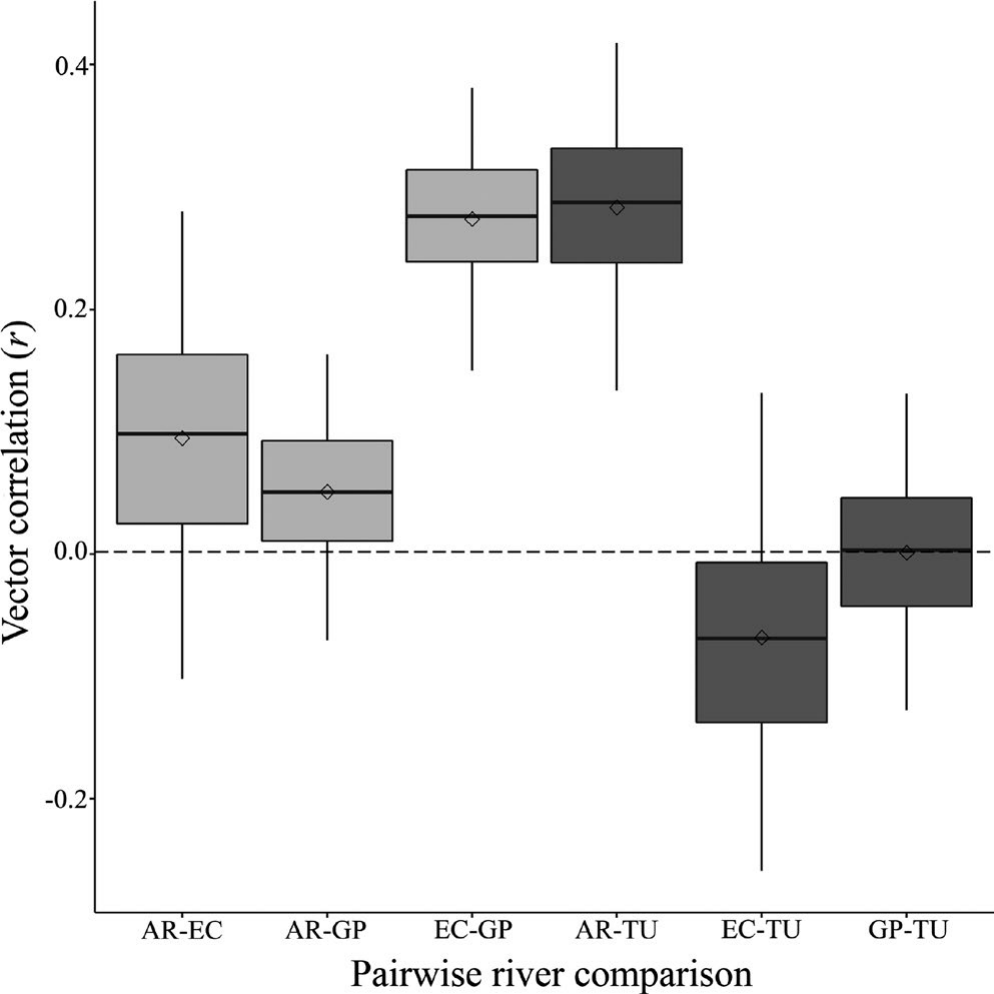
Correlations between pairs of rivers comparing the direction of evolution in color patterns within rivers from high‐predation sites to low‐predation sites. Pairwise river comparisons are represented by each box and whisker where river pairs are identified on the *x* axis. Boxes indicate the interquartile range and whiskers show the 95% confidence interval of the estimate for the correlation between vectors of direction of color evolution. Within each boxplot, the median and mean of the distributions are represented by the solid line and diamond, respectively. The horizontal dashed line represents the mean correlation between random vectors. The horizontal dotted line represents the mean expected correlation between pairs of rivers within the Caroni drainage system. The four rivers included in the pairwise comparisons are the Aripo (AR), El Cedro (EC), Guanapo (GP), and Turure (TU). AR, EC, and GP belong to the Caroni drainage and TU to the Oropuche; within‐drainage pairwise comparisons are shaded in light gray while between‐drainage comparisons are shaded in dark gray

The natural replication of the ancestor‐descendant relationship between rivers allows us to determine whether the direction of color pattern evolution within rivers is more similar between rivers than expected by chance. Figure 4 illustrates the similarity in direction of color evolution from the high‐predation to the low‐predation sites for each of the four rivers by showing the pairwise correlation between vectors that describe the average color pattern evolution within each river. Since we were unable to test this pattern for all four rivers at the same time, each pair of rivers (e.g., Aripo vs. El Cedro) is represented by a bootstrap distribution that shows the estimate of the correlation and the uncertainty in that estimate in the direction of evolution between rivers (95% Confidence Interval). Of the six pairwise comparisons, the El Cedro‐Guanapo (observed = 0.331, CI = 0.150–0.381) and the Aripo‐Turure (observed = 0.376, CI = 0.133–0.418) pairs were more correlated than expected by chance (mean correlation of random vectors = 0.002; Figure 4). The remaining four pairwise comparisons were not more correlated than expected by chance: Aripo‐El Cedro (observed = 0.105, CI = −0.102 to 0.280), Aripo‐Guanapo (observed = 0.062, CI = −0.070 to 0.163), El Cedro‐Turure (observed = −0.094, CI = −0.259 to 0.132), Guanapo‐Turure (observed = 0.006, CI = −0.127 to 0.131).

Of the four rivers included in our analysis of direction of color evolution, three rivers belonged to the Caroni (Aripo, El Cedro, and Guanapo) and one to the Oropuche (Turure) drainage. This allowed us to ask whether correlations in direction of color evolution between rivers belonging to different drainages were weaker than correlations within drainage. The cross‐drainage correlations were somewhat more variable than the within‐drainage correlations; interestingly, the cross‐drainage Aripo‐Turure correlation is just as strong as the strongest within‐drainage correlation (Figure 4).

### 3.3 Comparison of phenotypic variance in color between predation regimes

**FIGURE 5:**
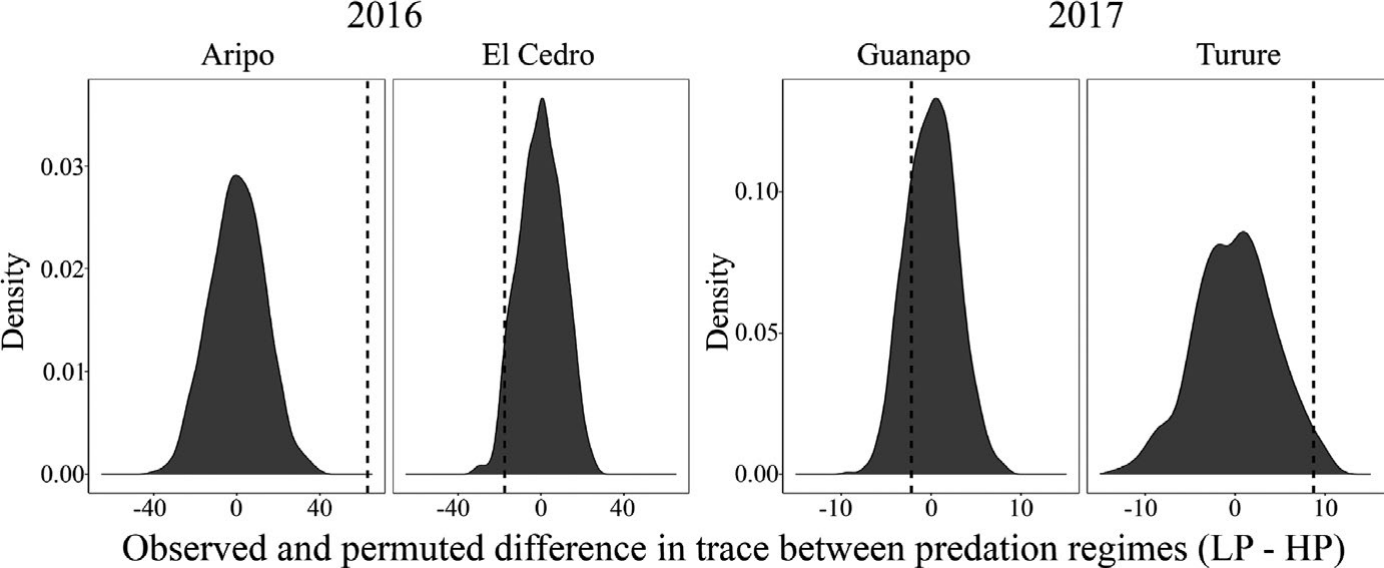
Observed (dashed line) and permuted (distribution) difference in total phenotypic variance (trace) between predation regimes where high‐predation (HP) values were subtracted from low‐predation (LP) values within a river

We next compared the total phenotypic variation between predation regimes for the four rivers for which both regimes were sampled in the same year, using the same data set. Figure 5 shows the results of the permutation tests of the difference in variance between high‐ and low‐predation populations within a river. In two rivers (Aripo and Turure), the low‐predation population had significantly higher total phenotypic variance (permutation *p*‐value <.001 and *p* = .009, respectively). In the other two rivers (El Cedro and Guanapo), the high‐predation population had nominally higher total phenotypic variance, but these differences were not significant (permutation *p*‐value = .099 and *p* = .113, respectively; Figure 5).

**FIGURE 6:**
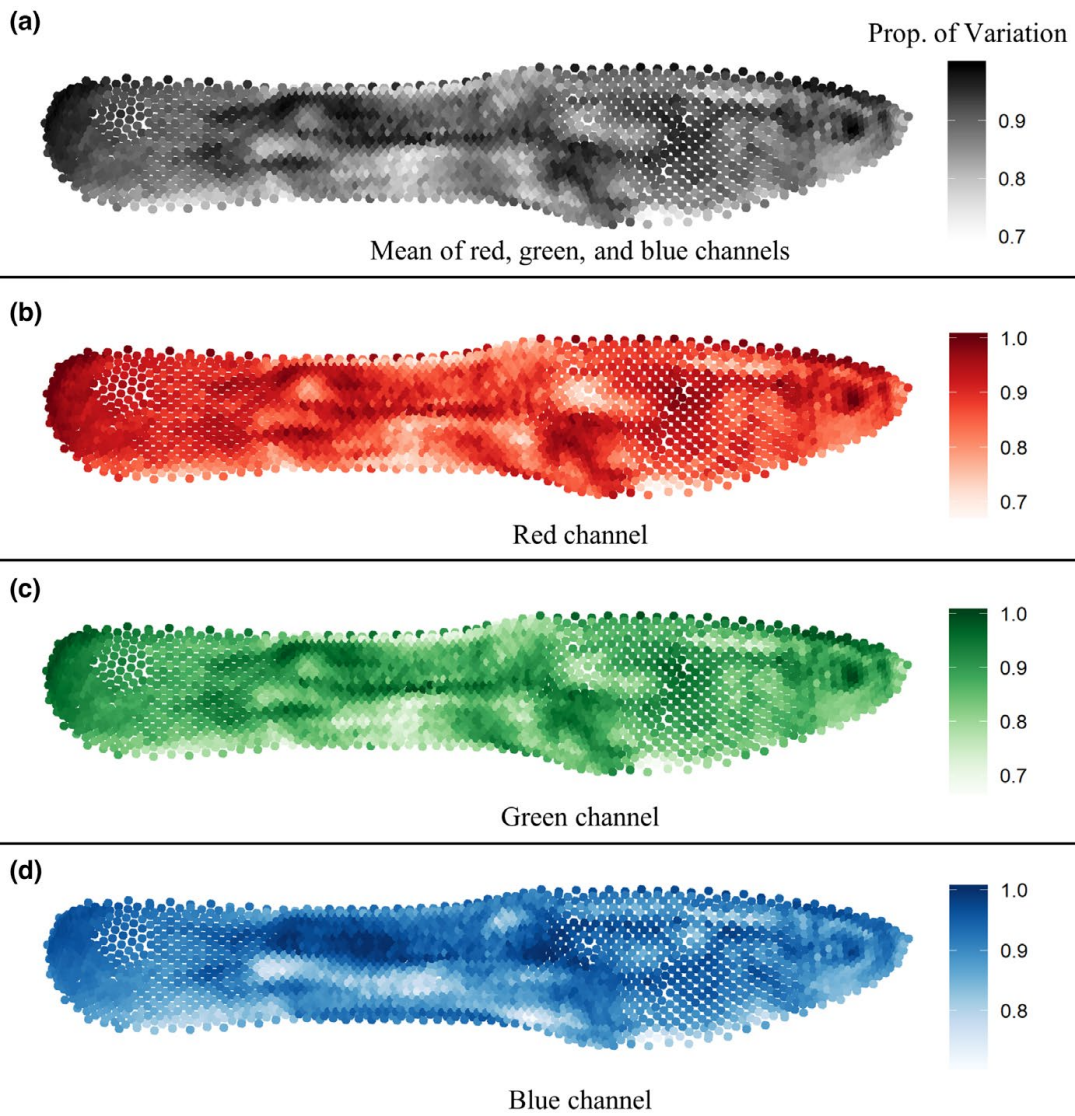
Heat maps showing the proportion of variation accounted for by differences within each of the 11 populations in color sampled at each of the 2,462 sampling points. Darker colors indicate higher proportion of variance distributed within, rather than among populations. Panel (a) shows the mean of the proportion of variation given by the red (b), green (c), and blue (d) channels

A spatially explicit analysis revealed the positions on the body that varied most in color within populations, relative to among‐population variance (Figure 6). For example, a position dorsal to the pectoral fin (“shoulder spot”) and several positions in the posterior region of the caudal peduncle were highly variable within populations. Conversely, in a region immediately posterior to the “shoulder spot,” most variation was distributed among, rather than within populations, as indicated by low values in the heat maps in Figure 6. Notably, these regions did not differ substantially in overall variance, suggesting that these patterns were driven by the distribution of variance within and among populations, and not by differences in general variability among body regions (Figure S2).

## 4 DISCUSSION

Our approach to quantifying variation in color patterns adds to the tools available to address questions requiring quantification of whole sample/organism color patterns. Our approach has four key advantages: it (a) allows the analysis of variation in chromatically and spatially complex patterns, (b) avoids loss of information by discretizing color or subjective identification of color elements, (c) allows high‐throughput analysis by using digital images of whole color patterns, and (d) provides flexibility with respect to sampling density and area.

We used this novel method to determine if it could accurately assign fish from 11 populations to the correct population and to evaluate whether the direction of color pattern evolution was consistent in four different rivers. We also asked if total phenotypic variation differed consistently between up‐ and downstream populations across drainages. Below, we discuss the performance of our approach on these tasks and compare our conclusions to those arrived at using different methods.

### 4.1 Multivariate classification and differentiation among populations

The guppy example highlights the usefulness of spatially explicit sampling of whole color patterns performed by *Colormesh*. Regardless of the type of specimen, our approach enables the use of color data to examine the granularity of whole‐pattern sampling, as well as identify regions within the pattern that contribute most to among‐population differentiation and within‐population variation. *Colormesh* allows the color of an individual to be characterized at different scales by using different numbers of points on the body and averaging over different numbers of pixels around each point. Variation in the number of semilandmarks would also influence the density of sampling points since these landmarks are used in the initial round of Delaunay triangulation. Therefore, an important precursor to analysis is to investigate how varying these parameters (rounds of triangulation, sampling circle diameter, number of semilandmarks) affects the conclusions of any analysis. For our guppy samples, increasing sampling density increased our ability to differentiate populations, and varying the number of pixels averaged around each point made little difference. This is likely because male guppy color pattern is complex, with color changing fairly dramatically at small spatial scales. Organisms with less fine‐grained patterns might be sufficiently sampled at coarser scales.

The discriminant analysis of principal components (DAPC) using our selected sampling scheme (4DT, 1 pixel diameter) separated populations largely by sampling year along the first discriminant function axis. Camera settings differed between years which likely accounts for the variation explained by this axis; however, since different populations were sampled in different years, this axis probably also captured some population differences in color. Although this year effect limited which downstream analyses we could perform, it also indicates that this method is sensitive to unsuspected sources of variation, which suggests that consistency in the image collection process is important.

DAPC also allowed us to identify axes of variation that consistently separated high‐ and low‐predation populations. Differences between predation regimes have been reported in previous studies assessing color patterns; however, the nature of these reported differences has varied. For example, in a study of 112 sites sampled from 53 streams located in Trinidad and Venezuela, Endler (1978) reported that males in high‐predation natural populations had smaller and fewer spots, and less coverage of bright (iridescent) colors. In contrast, Millar and Hendry (2012) reported few consistent differences between high‐ and low‐predation populations across five different river systems even though methods of color quantification (color categorization and measures of discrete color spots as judged by human vision) were similar to those used by Endler (1978). Investigations of color evolution across a similar ecological gradient, but over short time scales, have also produced varying results. Using populations translocated from high‐predation to low predation, and a combination of spectrometry, digital photography, and visual modeling, Kemp and colleagues reported that color evolved along different trajectories in the Aripo and El Cedro river introductions (Kemp et al., 2008, 2009). While there were differences in color quantification methods in these studies and ours, strong ecological correlates of color divergence suggest that these differences between river systems are driven primarily by biology, not methodology (Kemp et al., 2018; Millar et al., 2006; Millar & Hendry, 2012).

### 4.2 Direction of color evolution

With our guppy example, we sought to test whether the repeated transition from high‐ to low‐predation pressure among rivers resulted in parallel directions of color pattern evolution among several rivers. Using the known ancestor‐descendant relationship between high‐ and low‐predation populations, we calculated correlations in direction of multivariate color evolution between rivers. Our results were similar to those reported in two previous studies in that we found inconsistencies in the direction of color evolution among the rivers. In both previous studies, an increase or decrease in quantity and size of individual color pattern elements were compared to determine similarity in evolutionary trajectories (Kemp et al., 2009; Millar & Hendry, 2012). Our approach to sampling color using *Colormesh* compliments these studies by assessing total phenotypic variation across the entire fish while avoiding the potential constraints imposed by the use of human‐determined color categories. Three of the four rivers (Aripo, El Cedro, and Guanapo) in our comparisons belong to the same drainage system (Caroni), with the El Cedro being a tributary of the Guanapo. Comparisons between pairs of rivers within the Caroni drainage system showed that the El Cedro and Guanapo rivers were more similar in the trajectory of evolution than the other pairs of rivers, but the degree of similarity was small (vector correlation <0.3).

Between‐drainage correlations included comparisons of a single river of the Oropuche drainage (Turure) with each of the three rivers belonging to the Caroni drainage. These three correlation measures were highly variable. We expected the Turure‐Guanapo and Turure‐El Cedro comparisons to show greater correlation in direction of evolution than the Turure‐Aprio comparison because the low‐predation Turure population was originally a guppy‐free location where guppies from a high‐predation population in the Arima river (in the Caroni drainage, collected near the confluence with the Guanapo river) were introduced by Haskins in 1957 (Magurran et al., 1992; Shaw et al., 1992). Genetic evidence indicates the introduced guppies have successfully established themselves all along the low‐ to high‐predation reaches of the Turure and have interbred with native Turure populations located downstream (Becher & Magurran, 2000; Fitzpatrick et al., 2015; Shaw et al., 1992). Surprisingly, the between‐drainage system comparisons found the direction of color evolution between the Turure and Aripo was more similar to that expected between rivers within the same drainage. One possible explanation for this is that, in contrast to the other rivers used in this comparison, the large barrier waterfall between the low‐ and high‐predation populations within the Turure River has prevented the migration of high‐predation guppies upstream; the fish in this low‐predation location are exclusively descendants of the transplanted fish (Shaw et al., 1992). This suggests that some aspects of the environment, beyond predation regime, and/or stochasticity play an important role in the direction of the color evolution when considering the overall patterns.

In our study, the consistent pattern of separation between high and low‐predation populations found in the DAPC suggested that the direction of color evolution might be quite similar in different rivers. It is important to note that our approach sampled all aspects of color data obtained by the digital imaging process. It is possible that evolutionary trajectories of some aspect of color are indeed more parallel then the overall pattern shows. The completeness of our characterization of color patterns allows the user to restrict subsequent analyses to subsets of the color pattern hypothesized to correspond to more predictable differences among the groups under study; methods that only assess limited aspects of color pattern do not allow alternative characterizations to be investigated without recharacterizing the original images. The *Colormesh* approach enabled our analysis of the multivariate direction of evolution providing a quantitative measure of degree of similarity or parallelism in independent replicates using methods developed for addressing these questions and similar questions for other kinds of quantitative traits.

### 4.3 Total phenotypic variation

Using the guppy system example, we also evaluated differences in total phenotypic variation within and among populations. The processes that promote variation in male guppy color patterns have been investigated extensively (e.g., Brooks, 2002; Brooks & Endler, 2001; Farr, 1977; Houde & Endler, 1990; Hughes et al., 1999, 2013), and based on this literature, we predicted that low‐predation sites should exhibit more phenotype variation than high‐predation sites. We did find that the low‐predation Aripo and Turure populations had significantly greater variance in overall color than the high‐predation populations in those rivers; however, the trend (though not significant) was in the opposite direction for the El Cedro and Guanapo populations. This lack of consistently greater variation in low‐predation populations suggests that *A*. *hartii* does not inflict stronger NFDS on guppies in low‐predation populations, or that other ecological conditions interact with the NFDS imposed by predators and females to determine levels of within‐population variance in male color. Future investigations should assess density of potential predators and other ecological and genetic patterns (e.g., effective population size and migration rates) to provide more insight into the determinants of variation in this ecologically important trait.

We also identified spatial positions on the body that were characterized by high within‐population variation. Given the evidence for NFDS, future studies could examine whether spatial positions exhibiting high within‐population variance are subject to stronger selection than regions with lower variance. Such studies could determine if female‐imposed sexual selection (e.g., Valvo et al., 2019) or predator‐imposed natural selection (e.g., Fraser et al., 2013) is influenced by the spatial distribution of variable color pattern elements.

### 4.4 Summary, extensions, and conclusion

The use of *Colormesh* provides a novel and unsupervised approach to the analysis of complex color patterns. In applying this pipeline, we focused on population differences and on patterns associated with repeated evolutionary transitions to novel ecological conditions in guppies. We emphasize, however, that this method of quantifying color, which results in a standard multidimensional representation, can be used to address a wide variety of questions for biological samples ranging from histology preps to whole organisms and can be integrated or combined with other approaches to color and pattern analysis. For example, patterns of correlation in color across the body could be assessed by standard methods in spatial statistics (Schabenberger & Gotway, 2005; Wackernagel, 2013). These methods can identify regular patterns such as stripes and spots, and capture variation in this patterning among individuals, populations, or species. Our approach can be easily extended to more than three color channels (e.g., UV), to different color spaces (CIELAB, HSV, etc.), and to other features such as polarization by adding dimensions to the multivariate trait vector. The continuous representation of color could be used for image segmentation or disparity analysis, applying approaches available in *patternize* and *colordistance* (Van Belleghem et al., 2018; Weller & Westneat, 2019). Critically, the highly multivariate and spatially explicit data produced by this method could be integrated with visual models based on the biology of the receivers of visual signals, such as those incorporated in *pavo2* and the QCPA platform (van den Berg et al., 2020; Maia et al., 2019). Visual modeling could resolve the apparent contrast between our quantitative results (low to moderate parallelism in the evolution of color patterns across different river systems), and the results of other studies that find that rivers within the same drainage tend to exhibit parallel changes (e.g., Kemp et al., 2018). It is possible that only some aspects of color pattern are relevant to predation risk or attractiveness, while other aspects of color pattern detected by *Colormesh* are not relevant. The representation of color pattern from *Colormesh* could be used in future analyses to test which aspect of pattern are in fact relevant to these processes.

*Colormesh* can also be used for detailed investigation of the inheritance of the complex guppy color pattern (see Paris et al., 2021). Some of the genes responsible for coloration are located on the sex chromosomes with several of these genes being Y‐linked (Lindholm & Breden, 2002; Winge, 1927). The guppy color pattern is often described as being so variable that no two fish look alike; however, some aspects of the color pattern are highly heritable (Brooks & Endler, 2001b; Hughes et al., 2005) and some appear to be inherited patrilineally (Endler, 1978; Houde, 1997; Winge, 1927). The extent to which guppy color patterns are sex‐linked (as opposed to sex‐limited in expression), and how these patterns relate to chromosomal evolution, have been the subject of considerable recent interest (Charlesworth, 2018; Gordon et al., 2012, 2017; Paris et al., 2021; Wright et al., 2017). The methods described here could be deployed to determine whether a finite set of color patterns exist within a population and to quantify the number of such patterns when they exist. For example, rather than using human judgment, DAPC (described above) can be used to infer the number of clusters of similar color patterns within a population (Jombart et al., 2010). Although we focus on the complex color patterns of male guppies for our examples, the standardization of specimen images enables the *Colormesh* approach to be useful in investigating the inheritance of color patterns for a wide range of taxonomic groups.

Overall, the color sampling method performed by *Colormesh* is a novel approach for analysis of complex color patterns; analyses of guppy color patterns highlight the value of the approach. *Colormesh* offers unique capabilities that allow for the analysis of variation in color patterns regardless of whether pattern elements are clearly defined and complements, rather than replaces, existing methods of color pattern analysis.

## CONFLICT OF INTEREST

The authors declare they have no conflict of interest.

## PACKAGE DOWNLOAD

The *Colormesh* package is available for download at Github: https://github.com/J0vid/Colormesh. The code and example images to demonstrate the application of the *Colormesh* sampling approach is available to users at the Github site.

## AUTHOR CONTRIBUTIONS

**Jennifer J. Valvo:** Conceptualization (lead); formal analysis (equal); funding acquisition (supporting); methodology (equal); software (equal); writing‐original draft (lead). **Jose David Aponte:** Conceptualization (equal); software (equal). **Mitch J. Daniel:** Formal analysis (supporting); writing‐review & editing (supporting). **Kenna Dwinell:** Data curation (supporting). **Helen Rodd:** Data curation (supporting); funding acquisition (equal); methodology (supporting); writing‐review & editing (supporting). **David Houle:** Formal analysis (supporting); methodology (supporting); writing‐review & editing (equal). **Kimberly A. Hughes:** Conceptualization (equal); formal analysis (equal); funding acquisition (equal); methodology (equal); writing‐review & editing (equal).

## ETHICAL APPROVAL

The research presented was described in Animal Research Protocol No. 1442 approved on 29 October 2014 and Protocol No. 1740 approved on 16 October 2017, by the Animal Care and Use Committee of Florida State University. A preprint version of this manuscript was made available through BiorXiv (https://doi.org/10.1101/2020.07.17.205369).

## Supporting information

Fig S1

Fig S2

Fig S3

Fig S4

Appendix S1

## Data Availability

The data used in this study are available via Dryad at https://doi.org/10.5061/dryad.zgmsbccc1.
