# Supplementary figures and images for: Using Delaunay triangulation to sample whole‐specimen color from digital images
Source: Ecol Evol. 2021 Aug 20;11(18):12468–84. doi: 10.1002/ece3.7992 (PMC8462138; doi:10.1002/ece3.7992)

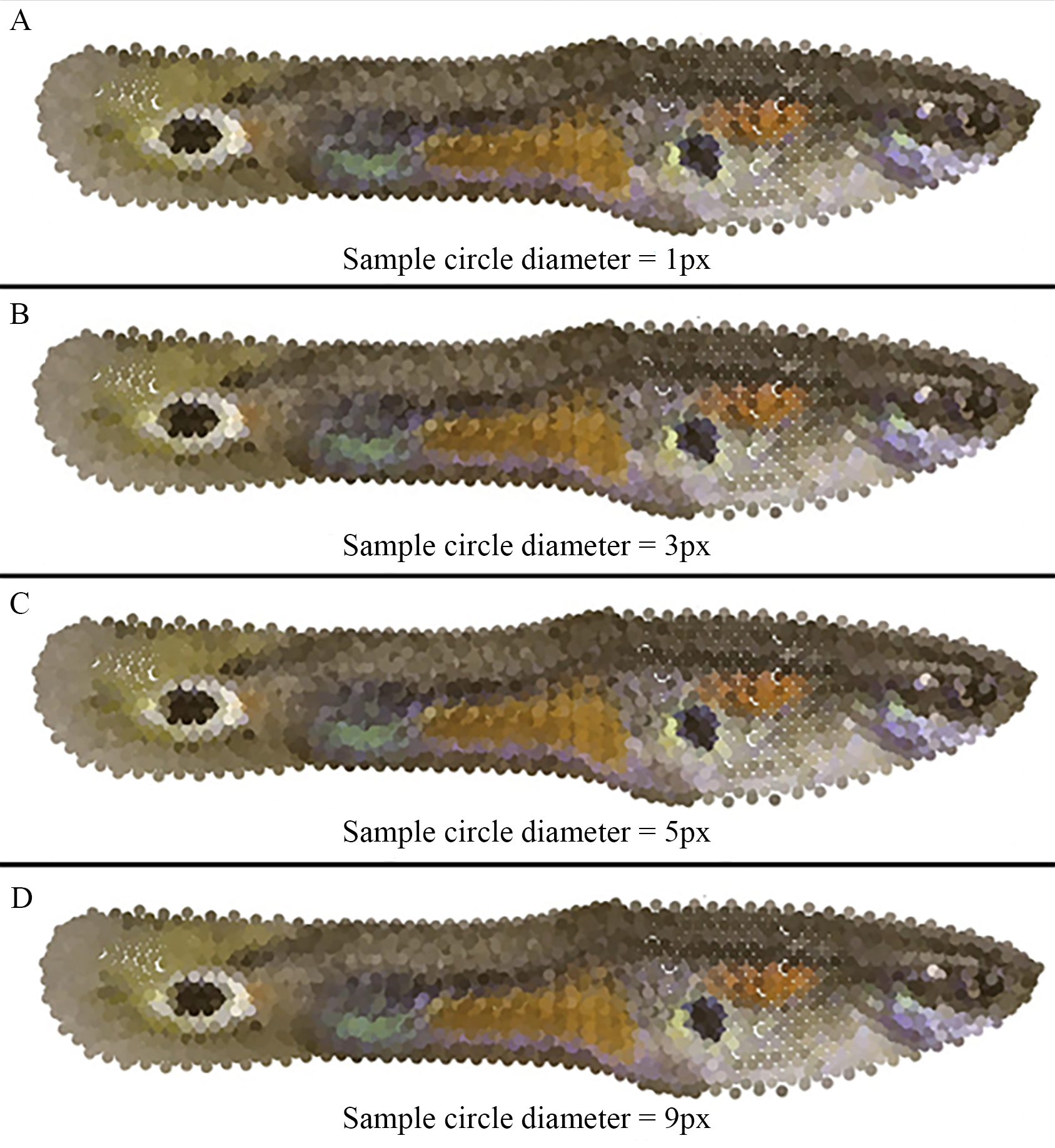

Supplement: Supplementary file 1 — Fig S1 [file ECE3-11-12468-s003.tif]

A

Coeff. of Variation (%)

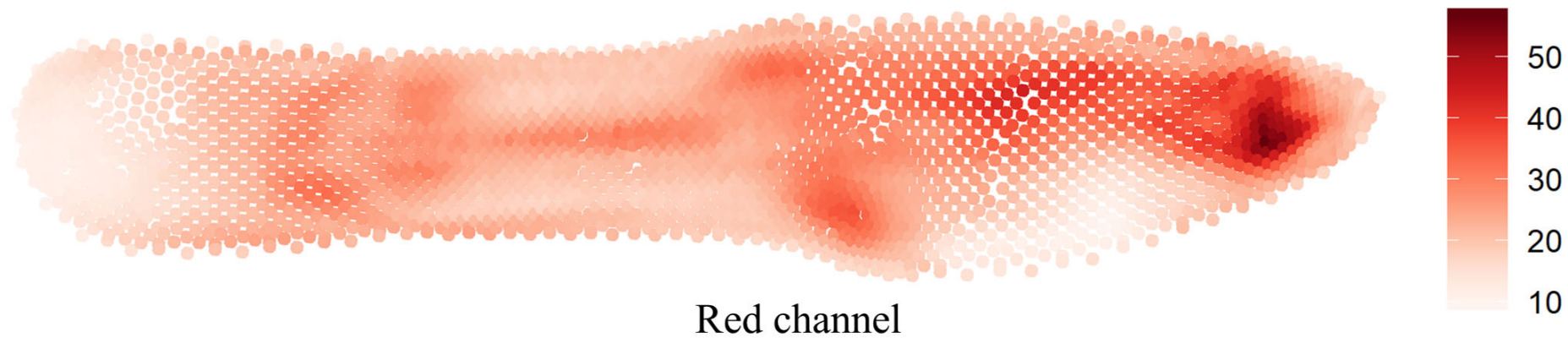

B

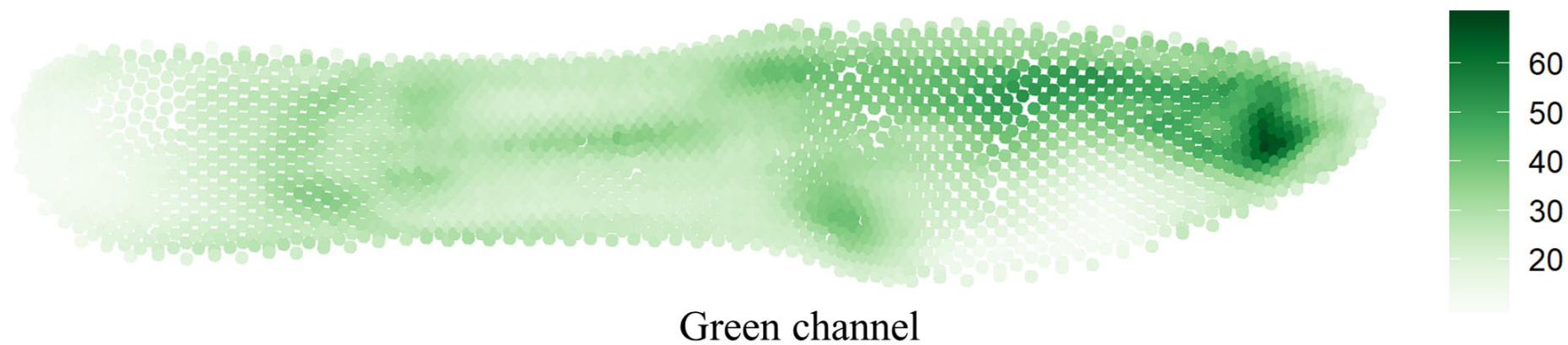

C

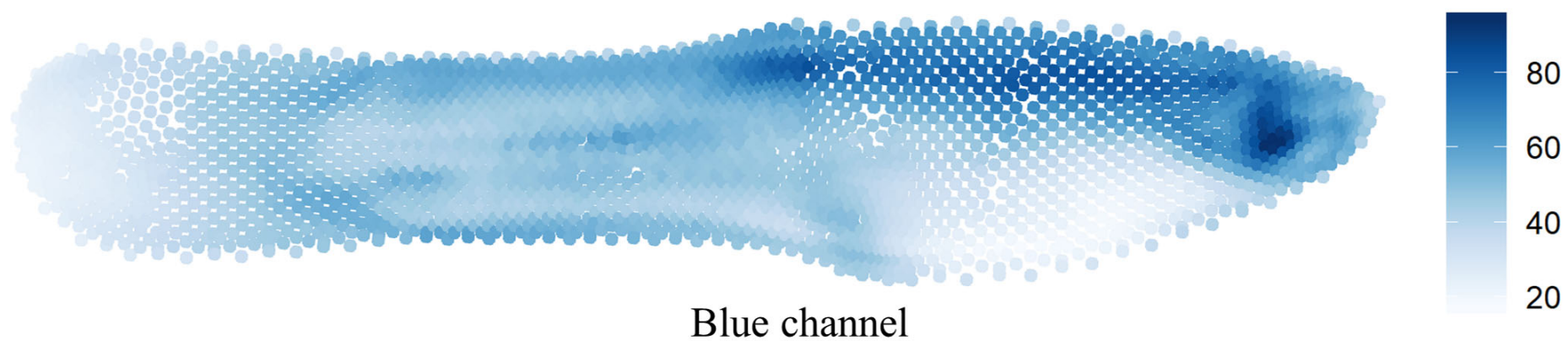

Supplement: Supplementary file 2 — Fig S2 [file ECE3-11-12468-s004.pdf]

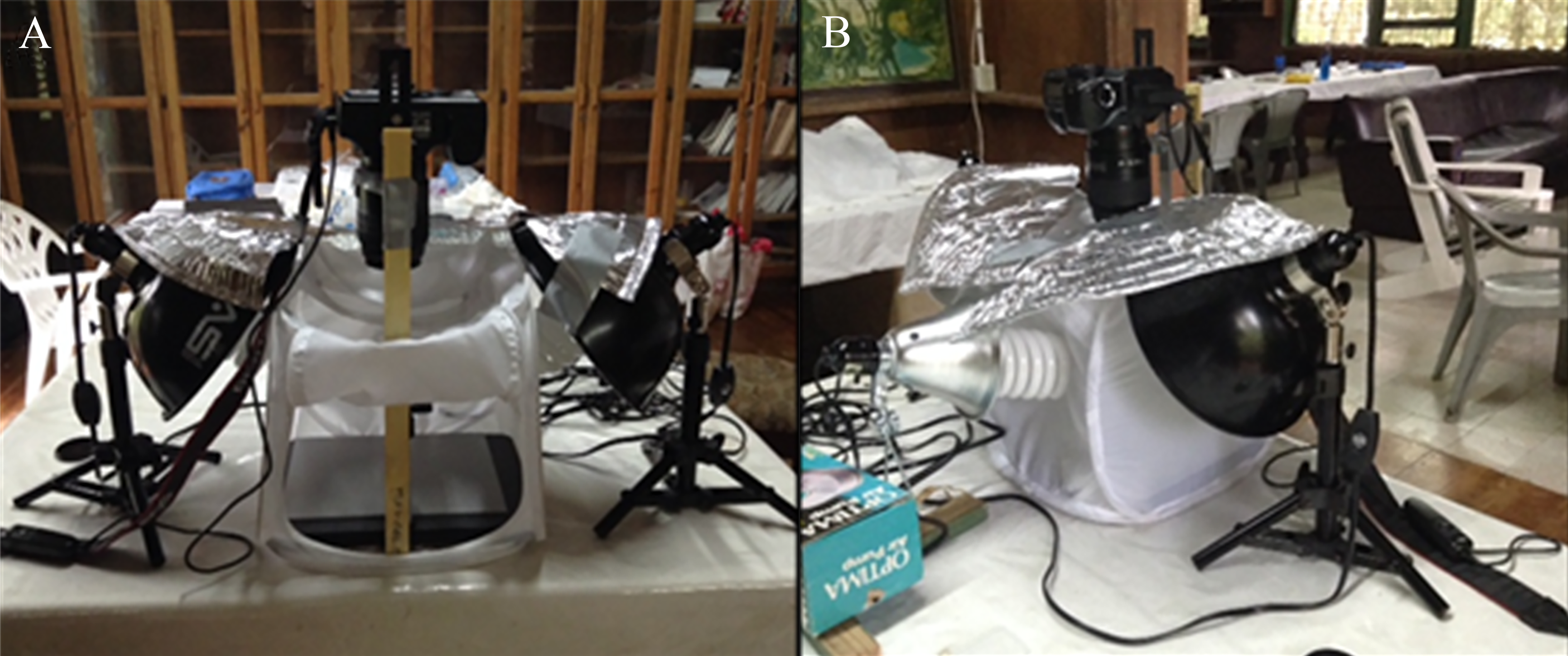

Supplement: Supplementary file 3 — Fig S3 [file ECE3-11-12468-s005.tif]

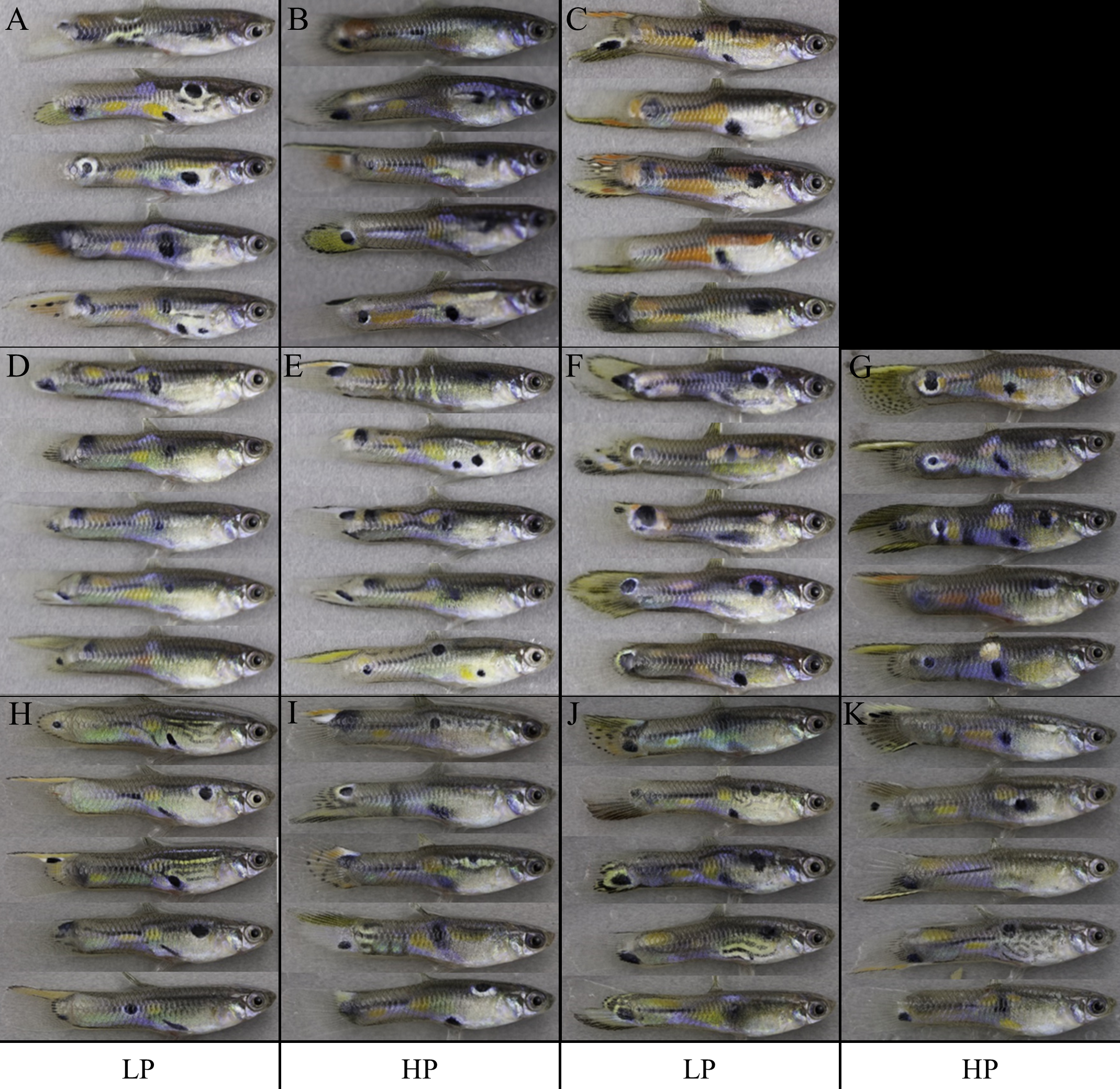

Supplement: Supplementary file 4 — Fig S4 [file ECE3-11-12468-s001.tif]
