# Supplementary material for: Using Delaunay triangulation to sample whole‐specimen color from digital images
Source: Ecol Evol. 2021 Aug 20;11(18):12468–84. doi: 10.1002/ece3.7992 (PMC8462138; doi:10.1002/ece3.7992)
Supplement: Supplementary file 5 — Appendix S1 [file ECE3-11-12468-s002.docx]

**Tables & Table Legends**

**Appendix Table S1.** River/tributary name(s) and predation regime, year in which males were collected, the total number of males sampled, river system drainage membership, and GPS coordinates of populations sampled.

| River/predation regime | Year | Total | Drainage | Latitude – N | Longitude - W |
| --- | --- | --- | --- | --- | --- |
| Aripo high-pred | 2016 | 57 | Caroni | 10.65474 | 61.22755 |
| Aripo low-pred | 2016 | 51 |  | 10.67123 | 61.22922 |
| El Cedro high-pred | 2016 | 47 | Caroni | 10.6567 | 61.26599 |
| El Cedro low-pred (Experimental) | 2016 | 54 |  | 10.663588 | 61.26584 |
| Guanapo high-pred (Twin Bridges) | 2017 | 57 | Caroni | 10.63989 | 61.24833 |
| Guanapo low-pred (Tumbasson) | 2017 | 45 |  | 10.70944 | 61.25778 |
| Marianne high-pred | 2017 | 24 | Northern | 10.76667 | 61.30000 |
| Marianne low-pred | 2016 | 36 |  | 10.75727 | 61.31523 |
| Paria low-pred | 2016 | 40 | Northern | 10.74740 | 61.26629 |
| Turure high-pred (Experimental) | 2017 | 40 | Oropuche | 10.65469 | 61.16946 |
| Turure low-pred (Experimental) | 2017 | 34 |  | 10.68606 | 61.17312 |

**Appendix Table S2.** Name, color code and RGB values for the Liquitex Heavy Body acrylic paint used for the color standards. RGB values for pixel colors range between 0 and 1, therefore RGB values were divided by 255 to determine the value to correct the measured values at each sampling point.

| Liquitex Color Name | Code | Red | Green | Blue |
| --- | --- | --- | --- | --- |
| Napthol crimson | 292 | 173/255 | 43/255 | 50/255 |
| Cadmium orange | 150 | 243/255 | 121/255 | 33/255 |
| Cadmium yellow medium | 161 | 254/255 | 244/255 | 17/255 |
| Emerald green | 650 | 43/255 | 163/255 | 73/255 |
| Ivory black | 244 | 26/255 | 13/255 | 21/255 |

**Appendix Table S3.** Below are the results from the *xvalDapc* function in the *adegenet* package using the number of PC’s associated with the lowest Root Mean Squared Error (RMSE) for each of the 12 different sampling schemes. Also provided is the proportion of successful placement into correct populations of the validation set and the number of PC’s retained. The sampling designs evaluated include all combinations of three sampling densities (2, 3, and 4 rounds of Delaunay triangulations (DT)) and four different sample circle sizes (diameter in pixels). RGB values of all pixels within sample circles having a diameter > 1 were average to provide a single R, G, and B value. Cross validations were performed on datasets where images from both years were combined as well as separate.

| Year | Circle size | 4DT | | | 3DT | | | 2DT | | |
| --- | --- | --- | --- | --- | --- | --- | --- | --- | --- | --- |
|  |  | Success | RMSE | PC’s | Success | RMSE | PC’s | Success | RMSE | PC’s |
| 2016 & 2017 | 1 | 0.91 | 0.11 | 100 | 0.90 | 0.12 | 100 | 0.85 | 0.17 | 100 |
|  | 3 | 0.92 | 0.10 | 150 | 0.92 | 0.10 | 100 | 0.86 | 0.16 | 100 |
|  | 5 | 0.93 | 0.10 | 100 | 0.90 | 0.11 | 200 | 0.87 | 0.15 | 100 |
|  | 9 | 0.92 | 0.10 | 150 | 0.91 | 0.10 | 150 | 0.88 | 0.14 | 150 |
| 2016 | 1 | 0.92 | 0.13 | 60 | 0.92 | 0.13 | 40 | 0.89 | 0.16 | 100 |
|  | 3 | 0.91 | 0.13 | 80 | 0.89 | 0.15 | 60 | 0.91 | 0.15 | 60 |
|  | 5 | 0.91 | 0.13 | 60 | 0.91 | 0.14 | 40 | 0.91 | 0.15 | 80 |
|  | 9 | 0.91 | 0.14 | 60 | 0.92 | 0.13 | 80 | 0.90 | 0.14 | 120 |
| 2017 | 1 | 0.93 | 0.11 | 40 | 0.90 | 0.15 | 40 | 0.83 | 0.22 | 60 |
|  | 3 | 0.90 | 0.14 | 60 | 0.89 | 0.15 | 60 | 0.81 | 0.23 | 20 |
|  | 5 | 0.91 | 0.15 | 100 | 0.88 | 0.17 | 80 | 0.82 | 0.23 | 60 |
|  | 9 | 0.93 | 0.12 | 80 | 0.89 | 0.15 | 80 | 0.86 | 0.20 | 40 |

**Appendix**

**Methods**

*Fish collection*

Fish were caught using butterfly nets and transported in sealed Nalgene bottles containing water with Stress Coat (API) to the nearby William Beebe Tropical Research Station, located in the lower Arima valley in the Northern Range, Trinidad. Fish were separated by population and maintained in single-sex 20-40L aquaria for 24-48 hours prior to taking their photographs. Water exchanges of 30% were performed daily using conditioned rainwater collected on site. Fish were fed Tetramin Tropical flake food twice daily.

*Image collection*

To compare color patterns, we collected digital photographs of male guppies (N=485) from the 11 populations (described in the *Methods: Populations sampled* section of this manuscript) by lightly anesthetizing them with buffered MS222. Males were placed on their side, dorsal side up, with the snout facing right, on a stage along with a scale and color standard. Using a camel-hair paintbrush, we positioned the dorsal fin and gonopodium away from the body of each fish and dabbed the fish body with a Kimwipe to remove excess water and reduce glare. They were photographed (Canon EOS 5D, 100mm f/2.8 macro lens) within a Photo Cube lighting tent to diffuse light and minimize glare. Three 65-watt daylight (white) compact fluorescent light bulbs (Fovitec StudioPRO, 5500K, full spectrum, Color Rendering Index = 90) in hooded light fixtures were positioned outside of the lighting tent. Two were positioned opposite each other, to the left and right of the photographer and angled down approximately 30 degrees from the plane of the camera lens (see Appendix Figure S3 for photography setup); the third light source was positioned on the side of the tent opposite from the photographer and angled down approximately 20 degrees. The configuration of the three lights minimized any shadow. Images were taken in RAW format and converted to TIFFs in Adobe Photoshop CC 2018. A minimum of two photos of each fish were collected and photographs of all males from the same population were taken during the same session. We photographed N=285 male guppies in 2016 and N=200 in 2017 for a total of N=485 images. All fish were photographed on an 18% grey background (Movo Photo Color/White Balance Card Set for digital photography). The photography equipment used in 2016 and 2017 was identical. However, the distance of the camera lens from the fish differed between years. We increased the distance between the stage and camera from 14.5cm in 2016 to 28cm in 2017 which allowed for the use of autofocus and a remote switch for a more consistent image quality. Because of this difference, we took year into account in subsequent analyses. For a sample of images from each of the 11 populations, see Appendix Figure S4.

*Image processing*

All data processing steps were performed on a Dell Precision with an Intel i7 CPU, Windows 10, and 16GB RAM. Images were processed using the *TPS Series* morphometrics software. Although the *Colormesh* package is capable of processing images, several other geometric morphometrics software are available and regularly used for shape analysis. Because users may already be familiar with landmark placement and unwarping with existing software, we designed *Colormesh* to allow for easy importation of processed images and landmark data for color sampling. *Colormesh* requires two image sets (a set of original images used in the calibration process and a set of consensus-shaped images) and their respective landmark coordinate data files. With the *TPS series* software, the final step in transforming images to the consensus shape is computer memory intensive which limits the amount of data that can be processed; to unwarp all 485 guppy images, we decreased the total data to be processed by using the batch cropping ability in Photoshop CC2018 to reduce individual image dimensions.

Using *tpsUtil* (V.1.78) we built the two TPS files for each of our populations’ image sets (cropped and original). Using the *tpsDig2* (V. 2.31) software, a lab assistant (K.D.) placed 62 landmarks around the perimeter of each fish within the set of cropped images. Seven traditional landmarks were first placed at the following locations: the tip of the snout, anterior and posterior connection points of the dorsal fin to the body, dorsal and ventral connection points of the caudal fin to the caudal peduncle, posterior and anterior connection points of the gonopodium to the body (Figure 1). K.D. then placed 55 additional semilandmarks between the traditional landmarks; the semilandmarks between each pair of traditional landmarks were approximately evenly spaced. All landmarks were placed in a counterclockwise direction (Figure 1). Using the original uncropped image sets, landmarks were also placed on each of the five colors in the color standard for use in calibration. To include the size standard with the appropriate TPS files for the cropped images, we used *tpsDig2* to calculate the image scale (pixels/cm) from the original uncropped images and added the scale information to each TPS file containing cropped images.

Populations were initially processed separately to generate population-specific consensus shapes using the *tpsRelw32* (v. 1.70) software (Rohlf 2018). Population-specific consensus shapes were determined prior to the overall consensus shape to avoid biasing fish shape toward a particular population since the number of fish sampled for populations varied. We saved the resulting landmark coordinates of the population-specific consensus shape into a new TPS file. To generate a consensus shape for all populations, we repeated this process using the TPS file we created that contained the landmark coordinates of the population-specific consensus shapes. The landmark coordinates of this overall consensus shape were then used as the target in the final step of image processing where images are unwarped to this shape.

The final step in image transformation was to “unwarp” and save images using *tpsSuper* (V. 2.05) (Rohlf 2015). The unwarping process transforms each subject to the overall consensus shape by mapping pixels from the consensus shape back to the corresponding pixels in the original image. Mapping pixels in this direction ensures all pixels from the original image are represented in the image that has been transformed to the consensus shape (Rohlf 2015). Because each image was unwarped to the same consensus shape and pixels are mapped from the consensus shape back to the original image, the pixel dimensions of the unwarped images are equal. At the completion of the unwarping process in *tpsSuper*, the unwarped image files were saved to be sampled using the *Colormesh* sampling pipeline.

Using the *Colormesh* pipeline, RGB color values were then extracted from each unwarped fish image. To calibrate these values, color corrections were applied based on color values extracted from the color standard in the original images. Because the known RGB values of the five colors included on the color standard were given on a scale of 0 to 255, values were divided by 255 to match the scale of values extracted by *EBImage* which range from 0 to 1 (Appendix Table S2).

To evaluate the consequences of using different sampling schemes the extraction of RGB color values was repeated for 12 different sampling schemes. We specified two, three, and four triangulations to provide sampling densities of 302, 842, and 2486 points, per fish. For each of these sampling densities, we evaluated four pixel diameters (one, three, five, and nine pixels). Sampling schemes were evaluated both when images from 2016 and 2017 were grouped together and separated (Appendix Table S3).

**A tutorial for using *Colormesh* to sample color from digital images**

The following example code will guide you through the process of using *Colormesh* to place landmarks, transform images to a consensus specimen shape, and extract color from these images. The process of using *Colormesh* is divided into three major sections below: Installation, Using *Colormesh*, and Extracting Your Data. The Using *Colormesh* section is further subdivided into several sections to explain processes such as preparing files, image processing (landmark placement and image transformation), color sampling, and calibration. Because some users may already be familiar with existing geometric morphometric software, we have enabled *Colormesh* to import files typically generated by external processing (e.g., TPS files). *Colormesh* can be used regardless of the level of image processing that has been completed externally. The example code below guides users through importing the required files needed for the Color Sampling Pipeline (Section 2.4). The external processing examples provided below used the *TPS Series* software by James Rohlf, available for free at the Stonybrook Morphometrics website (http://www.sbmorphometrics.org/).

1. Installation:

Installing *Colormesh* from github:

devtools**::**install_github**(**"https://github.com/J0vid/Colormesh"**)**

1. Using *Colormesh* (V2.0)

*Required files for Colormesh (V2.0) Color Sampling*

The files listed below are required to proceed with the Color Sampling Pipeline (Section 2.4). Some of the required files are obtained during image processing. Image processing may be completed entirely within the *Colormesh* package. Alternatively, some or all of the image processing steps may be completed externally in your geometric morphometric program of choice given landmark data are contained in a TPS file format. Required files are:

- A .CSV file containing factors such as the specimen image names - these names must be unique. The first column MUST contain the unique image name. This .csv file will be used as a check to ensure measured color and calibration correction (if used) are associated with the appropriate image. If image unwarping (to the consensus shape) was completed externally, include the unique image names of the unwarped images in the second column. Any additional columns containing factors needed for your organization or identification (e.g., population name) can be included after the image name column(s).
- A .CSV file containing the known RGB values of the colors on the color standard to be used for calibration. They should be on a scale of 0 to 1. Each row is a color on the standard, each column is a color channel; the know R, G, and B values must appear in columns 1, 2, and 3, respectively. If known RGB values are on a scale of 0-255, simply divide by 255 to convert values to the proper scale.
- Two image file folders: One file folder containing the original images that have the color standard and another file folder for the unwarped images. If unwarped images were generated externally, they can be stored in this unwarped file folder. if unwarping images within *Colormesh*, this folder will become populated with the unwarped images.
- Two landmark coordinate data arrays: one having coordinate data for landmarks placed on the color standard and the other having landmark data for the consensus shape of the specimens. If landmark placement and unwarping of specimen images is performed within *Colormesh*, these arrays will be generated when using the functions described below. If these landmark data files are generated externally, they're typically in the form of TPS files. These TPS files are easily loaded into *Colormesh* using a function that converts them into the appropriate array format (see below).

2.1 Preparing the required CSV Files

- Using base R, read in the .csv containing the specimen image names (omit file extensions such as .jpg or .tif) and identification information. The first column MUST contain unique image names. The remaining columns can contain any other information you may need to identify your specimens.
- Using base R, read in the .csv containing the known RGB values for each of the colors on your color standard. The color channel values should be on the scale of 0 to 1; if they are out of 255, simply divide by 255. The rows of this csv should equal the number of colors sampled from the color standard. Each column should provide the known color RGB values for each of the colors on the standard. For example, if you have 5 colors on the color standard, you will have 5 rows. The first column of the csv should contain the known RED color channel values for each of the five colors, the second column should contain the known GREEN color channel values, and the third column should have the known BLUE color channel values.

specimen.factors <- read.csv("C:/Users/jennv/Desktop/Colormesh_test_jpg/specimen_factors.csv", header = T)

known.rgb <- read.csv("C:/Users/jennv/Desktop/Colormesh_test_jpg/known_RGB.csv", header = T)

2.2 Image Processing: Landmark placement & generating consensus shaped images

2.2.1 Landmark placement

Landmark placement may be performed either within the *Colormesh* environment (Section 2.2.1.1, below) or externally (Section 2.2.1.2, below). The aim of landmarks placement is to generate the two arrays containing landmark coordinate data: one array having coordinate data for landmarks placed around each specimen and the other array having coordinate data for landmarks placed on the color standard. Landmarks placed within the *Colormesh* environment will automatically generate the appropriately formatted arrays. Alternatively, landmarks placed using other software that are in the TPS file format can simply be imported, as described in Section 2.2.1.2, below.

2.2.1.1 Landmark Placement within the *Colormesh* environment

*Colormesh* calls on the image digitization ability found in the *geomorph* package to create the required landmark data array. The *landmark.images* function behaves similarly to the *digitize2d* function within the *geomorph* package; it will temporarily convert images to jpgs solely for obtaining landmark coordinates. A plot window will open with the first image. If the user defined a scale (e.g., scale = 10), the user will be prompted to first set the scale; if no scale was defined, the user will begin placing landmarks. In the example code below, the scale = 10. To set the scale, the user will create a line segment that expands across 10mm of the scale. To draw the line segment, the user first aligns the crosshairs on the scale to where the first of two points will be placed. Click the left mouse button to place the first point of the line segment to be drawn. To place the second point, the user aligns the crosshairs on the scale at the distance defined in the function and clicks to place this point, drawing a line segment. The user will be prompted as to whether they would like to keep the scale - to redraw the line segment, type "n". To keep the segment, type "y". The user will now begin placing the landmarks around the specimen. Follow the prompts in the R console. After placement of each landmark, the user will be prompted as to whether they would like to keep the landmark - "y" will advance to the next landmark, "n" will allow the user to place that landmark again (the "old" landmark will appear on the specimen, however, the recorded coordinates of the old landmark are replaced with the new coordinates). IMPORTANT: Be sure you have entered a "y" before proceeding to the next landmark - omission of a landmark will require you to start over with ALL landmark placement. After placing the number of landmarks defined in the function (nlandmarks = ), the user is prompted to advance to the next specimen. Upon completion of landmark placement on all specimens, a TPS file will be written to the directory specified in the function and the array of coordinates will be stored in the R environment.

## The landmark.images function initiates the landmarking process. In this example, 62 landmarks are placed: 7 traditional landmarks and 55 semilandmarks.

specimen.LM <- landmark.images(imagedir = "C:/Users/jennv/Desktop/Colormesh_test_jpg/", image.names = specimen.factors[,1], nlandmarks = 62, scale = 10, writedir = "C:/Users/jennv/Desktop/Colormesh_test_jpg/", tps.filename = "specimen_LM.TPS")

## A new array is defined containing the coordinates of the landmarks placed on the color standard in each image. These coordinates identify where on the standard to sample the known color values that will be used during the calibration process.

calib.LM <- landmark.images(imagedir = "C:/Users/jennv/Desktop/Colormesh_test_jpg/", image.names = specimen.factors[,1], nlandmarks = 5, writedir = "C:/Users/jennv/Desktop/Colormesh_test_jpg/", tps.filename = "calib_LM.TPS")

2.2.1.2 External landmark placement imported into the *Colormesh* environment

Landmarks can be placed using your geometric morphometric software of choice that generates a TPS file. Two TPS files will need to be created and imported: one having the coordinate data for landmarks placed around each specimen and another TPS file where landmarks were placed on the color standard. The function *tps2array* will read in the .TPS file containing landmark coordinate data and convert the information into the required array format.

## For clarity, we added .ext in the example code below to identify these data as coordinates that were imported into the Colormesh environment.

specimen.LM.ext <- tps2array("C:/Users/jennv/Desktop/Colormesh_test_jpg/orig_LM_jpg.TPS")

## The code below reads in the TPS file containing the coordinates for landmarks placed on the color standard contained within each specimen image.

calib.LM.ext <- tps2array("C:/Users/jennv/Desktop/Colormesh_test_jpg/calib_LM_jpg.TPS")

2.2.2 Transforming images to a consensus shape within *Colormesh*

Similar to landmark placement, images can be unwarped to a consensus shape either within the *Colormesh* environment (described below) or in your favorite geometric morphometrics software then imported into Colormesh for sampling (Section 2.3, below). Here, we describe the use of the *tps.unwarp* function to transform images to a consensus shape. This process generates two of the required files needed as input for the *Colormesh* Sampling Pipeline (Section 2.4): the array of landmark coordinates of the consensus shape and the set of images where specimens have been unwarped to a consensus shape.

Images that are unwarped to a consensus shape within the *Colormesh* environment must be of the same pixel dimensions (height x width). For example, our images are 4368 pixels x 2912 pixels. Unwarping to a consensus shape within *Colormesh* is performed by the *tps.unwarp* function. The function first performs a Generalized Procrustes Analysis by employing the utilities of the *geomorph* package. Then, the *imager* package is used to perform a thin-plate spline (TPS) image transformation. Finally, the resulting unwarped images are saved as PNG image format files in the directory specified by the user.

2.2.2.1 Define perimeter map and sliding landmarks (if any)

The first step is to define the perimeter map of the specimen and identifying which landmarks, if any, are sliding landmarks (semilandmarks). This perimeter map tells *Colormesh* what order to read the landmarks so that a perimeter is drawn around the specimen in a "connect-the-dots" manner. This perimeter map is used in both the unwarping process for sliding landmarks and the Delaunay triangulation (described below) to determine sampling locations. In the guppy example below, the first seven landmarks that were placed around the guppy are the traditional landmarks (placed at locations that are easily identifiable among images); the remaining 55 landmarks are referred to as semilandmarks. Semilandmarks are interspersed between the traditional landmarks and allowed to slide along the tangent of the curve they create when generating a consensus shape. The *make.sliders* function identifies which landmarks are traditional landmarks, and therefore will not slide in the calculation.

## Define perimeter map (order the points occur around the perimeter)

perimeter.map <- c(1, 8:17, 2, 18:19, 3, 20:27, 4, 28:42,5,43:52, 6, 53:54, 7, 55:62)

## Define sliders (main.lms = identifies which of all 62 landmarks are the traditional landmarks and therefore will NOT slide)

sliders <- make.sliders(perimeter.map, main.lms = 1:7)

2.2.2.2 Calculating the consensus shape

The second step is to calculate the consensus shape of the specimens. Prior to running the *tps.unwarp* function, you will need to create a file folder as a destination for the function to write the unwarped images. The information required by the function includes: the directory containing the original specimen images that are to be unwarped to the consensus shape identified by the "imagedir" argument (note: these images must all have the same pixel dimensions). Also provided to the function are the landmark coordinate data array for the landmarks that were placed around each specimen contained in these images. To associate the coordinate data with the appropriate images, you must provide the image names from the CSV file (1st column). If you have defined landmarks that are semilandmarks, and therefore allowed to slide, they also need to be identified. And finally, you must provide the directory where *Colormesh* will write the unwarped images. These unwarped images will be saved as PNG images, which is an uncompressed (lossless) image format.

## The example code below defines the landmark coordinate array generated in Section 2.2.1.1 (above)

unwarped.jpg <- tps.unwarp(imagedir = "C:/Users/jennv/Desktop/Colormesh_test_jpg/", landmarks = specimen.LM, image.names = specimen.factors[,1], sliders = sliders , write.dir = "C:/Users/jennv/Desktop/Colormesh_test_jpg/unwarped_images_jpg/")

## The example code below defines the landmark coordinate array generated in Section 2.2.1.2 (above) (see landmarks = specimen.LM.ext)

unwarped.jpg <- tps.unwarp(imagedir = "C:/Users/jennv/Desktop/Colormesh_test_jpg/", landmarks = specimen.LM.ext, image.names = specimen.factors[,1], sliders = sliders , write.dir = "C:/Users/jennv/Desktop/Colormesh_test_jpg/unwarped_images_jpg/")

The output of the function is a list having two elements. The "$target" element of the list is the landmark coordinate data for the consensus shape generated by the function. The names given to the unwarped images appear as the 2nd list element. The resulting unwarped images are written to the directory given by the user; these images are sampled in the Color Sampling Pipeline (Section 2.4, below). When image files are opened, specimens will now have the same shape. Note: Some black areas near the edges of the images are expected as they are part of the unwarping process.

2.3 Image Processing was performed externally - importing the required files

If the entirety of image processing (Landmark placement and unwarping to a consensus shape) are performed externally, *Colormesh* can import all of the required files for the Color Sampling Pipeline (section 2.4). Below, we identify the required information to prepare for color sampling. This includes:

- Defining the perimeter map to be used when generating the sampling template (Delaunay triangulation).
- The specimen factors CSV: the unique unwarped image names must appear in the 2nd column; original image names appear in the 1st column.
- The CSV having the known RGB values of the color standard.
- The two required arrays containing landmark coordinate data: the coordinates of landmarks placed on the color standard and the other array will be the landmark coordinate data of the CONSENSUS shape. These are imported using the *tps2array* function described above (Section 2.2.1.2).
- The two required images sets residing in their own folders. One image set is the original images (with the color standard) and the other image set is the unwarped images.

## Defining the perimeter map - this will be used in the Color Sampling pipeline. This is the order of the row of x,y coordinates that will connect the landmarks in a "connect-the-dots" manner

perimeter.map <- c(1,8:17,2, 18:19,3,20:27,4, 28:42,5,43:52,6,53:54,7,55:62)

## Example code for reading in the two CSV files

## NOTE: First column = original image names, 2nd column = unwarped names

specimen.factors.ext <- read.csv("C:/Users/jennv/Desktop/Colormesh_test_jpg/specimen_factors_ext.csv", header = T)

known.rgb <- read.csv("C:/Users/jennv/Desktop/Colormesh_test_jpg/known_RGB.csv", header = T)

## Example code for converting TPS files to the appropriate array format

## NOTE: CONSENSUS SHAPE COORDINATES ONLY

consensus.LM.ext <- tps2array("C:/Users/jennv/Desktop/Colormesh_test_jpg/consensus_LM_coords.TPS")

calib.LM.ext <- tps2array("C:/Users/jennv/Desktop/Colormesh_test_jpg/calib_LM_jpg.TPS")

## Create two image folders holding the two sets of images (the original images set for the calibration process and the unwarped image set for the Color Sampling pipeline).

2.4 Color Sampling Pipeline

To proceed with color sampling, you should now have available to *Colormesh*:

- The two required CSV files (image information and known RGB values of the standard).
- The two landmark coordinate arrays: one having landmark coordinate data of the CONSENSUS SPECIMEN SHAPE and the other having the landmark coordinate data of where to sample the color standard for the calibration process.
- Two sets of images located in their own directories: the set of images that were unwarped to the consensus shape and the original set of images containing the color standard.

In the Color Sampling pipeline, there are two main processes: 1) defining the sampling template (i.e., sampling density) and 2) defining the sampling circle size and measuring RGB values. For each of the processes, we have included several checks along the way. These include alignment checks to confirm the orientation of the image during the sampling process and overlapping of sampling circles. In addition, we provide several options for visualizing your plots under each section.

2.4.1. Calculating the sampling template (sampling density)

*Colormesh* uses Delaunay triangulation as an unsupervised method of determining locations to sample color from the consensus shaped specimen images. The first round of Delaunay triangulation uses the landmark coordinates of the consensus shape as the vertices of the triangles. It reads in the landmark coordinates of this consensus based on the order defined in the *perimeter.map* variable. The function that creates this mesh was designed to provide the user with flexibility in sampling density based on the number of rounds of triangulation specified by the user; more rounds provide a greater density of sampling points.

2.4.1.1 Generating the sampling template and checking alignment

The sampling template is generated by the *tri.surf* function and is an integer defined by the user. The *tri.surf* function calculates the X,Y coordinates of the centroid for each triangle generated by Delaunay triangulation; *Colormesh* calls on the *tripack* package to perform the Delaunay triangulation. If more than one round of triangulation is specified by the user, these centroids function as vertices for subsequent rounds of triangulation. At the completion of the user-specified rounds of triangulation, the pixel coordinate for each triangle's centroid is saved as sampling coordinates. The arguments defined in the function include: the array having the coordinates of the **consensus shape**, the perimeter map, a test image to check the alignment of the sampling template, and a logical argument to address whether to flip the y-coordinates (see below). By default, flip.delaunay = FALSE. Be sure your specimen.sampling.template is defined with the correct orientation (indicated by whether the triangulation overlay is properly aligned). The alignment check draws a yellow line around the perimeter of your specimen and red circles are plotted at the pixel coordinates that will be sampled (NOTE: circles are sized to be easily visible and do not represent the number of pixels that will be sampled).

IMPORTANT: Test that your sampling points properly overlay your image. Image readers (e.g., EBImage & imager) place the 0,0 coordinate in the upper left corner. In contrast, the coordinates in the TPS file place 0,0 in the bottom left corner. *Colormesh* assumes this to be true. The example code below demonstrates how to load a test image and plot the sampling template over the image to check alignment.

## Reading in a test image using the imager package

align.test1 <- load.image("C:/Users/jennv/Desktop/Colormesh_test_jpg/unwarped_images_jpg/IMG_7658_unwarped.png")

## In the examples below, num.passes = 3 means three rounds of Delaunay Triangulation will be performed.

## Below shows example code using the consensus shape array that was calculated by the tps.unwarp function where unwarping was done within the Colormesh environment(Section 2.2.2, above). When flip.delaunay = F, the template was not aligned correctly; the tri.surp function was re-ran with flip.delaunay = T to define the specimen.sampling.template with the correct orientation.

specimen.sampling.template <- tri.surf(tri.object = unwarped.jpg$target, point.map = perimeter.map, num.passes = 3, corresponding.image = align.test1, flip.delaunay = F)

specimen.sampling.template <- tri.surf(tri.object = unwarped.jpg$target, point.map = perimeter.map, num.passes = 3, corresponding.image = align.test1, flip.delaunay = T)

## Below shows the example code if you imported the consensus specimen shape from a TPS file and converted it to an array (Section 2.3 above).

specimen.sampling.template <- tri.surf(tri.object = consensus.LM.ext, point.map = perimeter.map, num.passes = 3, corresponding.image = align.test1, flip.delaunay = T)

2.4.1.2 Visualizing the sampling template

We have included the ability to plot the sampling template generated by the *tri.surf* function. The example code below shows how to plot the template where the specimen will be sampled. You may specify the style = "points" to plot the location of the all the points (perimeter and interior) that will be sampled, style = "perimeter" will print only the perimeter points, style = "interior" will plot only interior points, and style = "triangulation" will plot the triangulation that was generated and the centroids of each triangle. For style = "triangulation" you may change the color of the triangles that were generated (wireframe.color = ), as well as the color of the centroid (point.color = ).

## Plotting a map of all points (both the perimeter and interior) that will be sampled

plot(specimen.sampling.template, style = "points")

## Plotting only the perimeter points

plot(specimen.sampling.template, style = "perimeter")

## Plotting only the interior points

plot(specimen.sampling.template, style = "interior")

## Plotting the map of the Delaunay triangulation and the centroids of the triangles

plot(specimen.sampling.template, style = "triangulation", wireframe.color = "black", point.color = "red")

The "triangulation" style can be plotted overlaying the *align.test1* image (defined above). The following code shows how to make this plot. The default colors for both the "triangulation" and "overlay" styles draw the triangles in black and the sampling points (centroids) in red. However, The user can change the color of the triangles and centroids using the point.color = and wireframe.color = arguments.

plot(specimen.sampling.template, corresponding.image = align.test1, style = "overlay", wireframe.color = "grey", point.color = "yellow" )

2.4.2 Setting the sampling circle size and measuring RGB

2.4.2.1 Checking for overlapping sampling circles

Because sampling circle size is controlled by the user, we offer a diagnostic tool with the function *point.overlap*. The example code below demonstrates the use of this function to determine whether sampling circles of a given pixel radius (px.radius = ) will overlap. For example, a sampling circle with px.radius = 2 will have a sampling circle diameter of 5 pixels; the radius is 2 pixels out from the centroid pixel defined by the sampling template. If the sampling template (defined in Section 2.4.1) is dense, this may result in the overlap of sampling circles depending on their size. This function checks for overlap of sampling circles and produces a data frame with the sampling point ID and the distance between the centroid pixels of those that overlap. This function also produces a plot showing sampling circles that overlap in red (Note: the circles of the plot are not drawn to scale).

overlap = point.overlap(delaunay.map = specimen.sampling.template2, px.radius = 2, style = "points")

2.4.2.2 Measuring RGB values

The *rgb.measure* function measures the RGB values of the points sampled from the unwarped specimen images (at the points identified above in the *tri.surf* function). To control the size of the sampling circle, the user provides the radius length (in pixels) out from the centroid, from which to sample the surrounding pixels. In this function, the user first provides the file path to the folder containing the unwarped (to the consensus shape) images that are to be sampled, followed unwarped image names, next is the "specimen.sampling.template" (which provides sampling coordinates), an integer for the user-specified size of the sampling circle **radius** in pixels (px.radius = 0 will only sample the centroid pixel), and the logical argument for whether you would like to apply the linear transform (based on international standard IEC 61966-2-1:1999),to convert sRGB values to linearized values.

## The example code below uses the unwarped image names generated within Colormesh by the tps.unwarp function (Section 2.2.2, above)

## NOTE: We use the specimen.sampling.template defined by 3 rounds of Delaunay triangulation below

uncalib_RGB <- rgb.measure(imagedir = "C:/Users/jennv/Desktop/Colormesh_test_jpg/unwarped_images_jpg/", image.names = unwarped.jpg$unwarped.names, delaunay.map = specimen.sampling.template, px.radius = 2, linearize.color.space = FALSE)

## If unwarped images were generated externally, the image names will come from the 2nd column of the csv file

uncalib_RGB <- rgb.measure(imagedir = "C:/Users/jennv/Desktop/Colormesh_test_jpg/unwarped_images_jpg/", image.names = specimen.factors[,2], delaunay.map = specimen.sampling.template, px.radius = 2, linearize.color.space = FALSE)

## If the color values of the image are in sRGB color space, the values can be linearized setting linearie.color.space = TRUE

linear_uncalib_RGB <- rgb.measure(imagedir = "C:/Users/jennv/Desktop/Colormesh_test_jpg/unwarped_images_jpg/", image.names = unwarped.jpg$unwarped.names, delaunay.map = specimen.sampling.template, px.radius = 2, linearize.color.space = TRUE)

2.4.2.3 Visualizing the sampled color

The example code below will plot the color sampled using the *rgb.measure* function. The "individual = " argument allows you to plot a specific specimen. The default of style = "points" which plots the color values that were sampled from the image (perimeter and interior). Similar to the plotting options above, you have the option of only plotting the perimeter or the interior points. To compare your plotted sampled color values to the original image the color values were sampled from, set style = "comparison". Note that a plot of sampled values where linearize.color.space = TRUE will be **darker than the original** image due to the application of the linear transform.

## Plotting measured color at all points

plot(uncalib_RGB, individual = 8, style = "points")

## Plotting measured color at only the perimeter points

plot(uncalib_RGB, individual = 8, style = "perimeter")

## Plotting measured color at only the interior points

plot(uncalib_RGB, individual = 8, style = "interior")

## Plotting measured color at all points along with the image the from where color was sampled

plot(uncalib_RGB, individual = 8, style = "comparison")

2.5 Color calibration

Color information across images can be pretty noisy due to inconsistent lighting, different camera settings, movement of the object, etc. We highly recommend adjusting for those differences by including a color standard in each image. Using the differences in color standard values between images to mitigate variation due to noise, landmarks placed on the color standard are used to sample known RGB values and adjust the sampled color of your specimen by the average deviation in each color channel.

*Colormesh* uses the coordinates of landmarks placed on the standard in each image to sample known color values. Prior to calibration, it is important to check the alignment of the sampling coordinates and the images. Once you have determined whether an alignment correction must be made, the *rgb.calibrate* function can then be used to correct each image's measured RGB values. The function samples the color standards of each image at the coordinates supplied by the calibration array. An image-specific color correction vector is calculated based on the mean deviation of each color channel from the known RGB values of the color standard in that image. The correction vector is then applied to the measured RGB values of each image.

2.5.1 Checking the alignment for sampling

Prior to calibrating each image, it is important to check that the sampling locations align with the color standard in the image. The code below plots colored dots at the locations where color will be sampled in the image. The user has the option to change the size and color of the dots that are plotted. This is a simple test to confirm the y-axis coordinates are correct. In the example below, yellow points are plotted over the locations that will be sampled for color calibration.

## Plot a test image to check that the landmark coordinates are aligned correctly over the standard. We specified the point color and size to make them visible

calib.plot**(**imagedir **=** "C:/Users/jennv/Desktop/Colormesh_test_jpg/", image.names **=** specimen.factors**[** ,1**]**, calib.file **=** calib.LM, individual **=** 3, col **=** "yellow", cex **=** 1**)**

2.5.2 Calibrating the measured RGB values

For the *rgb.calibrate* function, the user first provides the name of the data that is to be calibrated, for example, "uncalib_RGB". Then the user provides the file path to the folder containing the original images (imagedir =). Next, "image.names = " is defined by providing the column containing the calibration image names from the csv containing this information. The coordinates of where to sample the color standard are defined as "calib.file = ". The logical argument for "flip.y.values" is available if the test image that is plotted shows that the y-coordinates need to be corrected (determined in the previous step with the calib.plot function). Finally, "color.standard.values = " is defined as the csv containing the known RGB values for the color standard. By default, the sampling circle that samples each color standard has a default radius = 2 pixels. You can change the size of the sampling circle with an integer when defining "px.radius = " as shown in the example code below.

*Note: If the *calib.plot* function showed proper alignment, set flip.y.values = F*

calib_RGB **<-** rgb.calibrate**(**uncalib_RGB, imagedir **=** "C:/Users/jennv/Desktop/Colormesh_test_jpg/", image.names **=** specimen.factors**[** ,1**]**, calib.file **=** calib.LM.ext, flip.y.values **=** F, color.standard.values **=** known.rgb**)**

## By default, the radius of the sampling circle is = 2. The user can change the sampling circle size by providing a different integer.

calib_RGB **<-** rgb.calibrate**(**uncalib_RGB, imagedir **=** "C:/Users/jennv/Desktop/Colormesh_test_jpg/", image.names **=** specimen.factors**[** ,1**]**, calib.file **=** calib.LM.ext, flip.y.values **=** F, color.standard.values **=** known.rgb, px.radius **=** 3**)**

To calibrate measured RGB values where linearize.color.space = TRUE, the *rgb.calibrate* function is used in the same manner. The *rgb.calibrate* function detects that this data was linearized because the logical in the list produced from the *rgb.measure* function = TRUE. When detected, both the known RGB values and the color measured from the color standard will be linearized prior to calculating the mean deviation from the known RGB values. This linearized color correction will then be applied to the linearized values collected from the specimen images.

linear_calib_RGB **<-** rgb.calibrate**(**linear_uncalib_RGB, imagedir **=** "C:/Users/jennv/Desktop/Colormesh_test_jpg/", image.names **=** specimen.factors**[** ,1**]**, calib.file **=** calib.LM.ext, flip.y.values **=** F, color.standard.values **=** known.rgb**)**

2.5.3 Visualizing the calibrated color

To plot your calibrated colors, you have the same options as above. With style = "points" both perimeter and interior points where color has been calibrated will be plotted. To print just the perimeter, style = "perimeter". With style = "interior" only the interior calibrated color values will be plotted. The **exception** is with the comparison plot. In the comparison plot, it compares the calibrated points to the uncalibrated points when style = "comparison". Linearized values can be plotted, as well; **Note**: Linearized RGB values will have a darker appearance.

## Plotting calibrated color values with style = "points"

plot**(**calib_RGB, individual **=** 5, style **=** "points"**)**

## Plotting calibrated color values with style = "perimeter"

plot**(**calib_RGB, individual **=** 5, style **=** "perimeter"**)**

## Plotting calibrated color values with style = "interior"

plot**(**calib_RGB, individual **=** 5, style **=** "interior"**)**

## EXCEPTION: This plot compares uncalibrated and calibrated color values

##Plotting calibrated color values with style = "comparison"

plot**(**calib_RGB, individual **=** 5, style **=** "comparison"**)**

3. Extracting your data

We created a simple function, *make.colormesh.dataset*, to compile your data into a single data frame. The user specifies which dataset they would like to include, the csv containing the specimen information, and lastly, a logical argument (TRUE/FALSE) as to whether perimeter point data is included.

This data frame will give individual specimens in rows. It will combine the image information csv file to the beginning of the data set. Following these columns, the measured values specific to each sampling point will be provided. After these columns, the x,y coordinate of each sampling point will be given.

##

final.df.uncalib **<-** make.colormesh.dataset**(**df **=** uncalib_RGB, specimen.factors **=** specimen.factors, use.perimeter.data **=** T**)**

## If you would like to write this datafram to a .csv file, include the file path where you would like the file to be saved following the write2csv argument.

final.df.uncalib.saved **<-** make.colormesh.dataset**(**df **=** uncalib_RGB, specimen.factors **=** specimen.factors, use.perimeter.data **=** T, write2csv **=** "C:/Users/jennv/Desktop/Colormesh_test_jpg/colormesh_data_uncalib.csv"**)**

**Appendix Figure S1.** Panels A – D show RGB color sampled using the four different sampling circle diameters. The density of sampling points in each panel was determined using four Delaunay triangulations. Panel (A) reproduced the RGB values samples from the 1 pixel (px) located at the centroid of each triangle. Panels B-D display the color produced from the mean red, green, and blue values of each pixel within the sampling circle of diameter = (B) 3 pixels, (C) 5 pixels, and (D) 9 pixels. For each of the four different sampling circle sizes, RGB values sampled at each location were plotted using the *plot* function in R. Plotted point size was equal (cex = 1.2) and selected to minimize white space between plotted points.

**Appendix Figure S2.** Heat maps showing the coefficient of variation (%) at each of the 2,462 sampling points for the (A) red, (B) green, and (C) blue color channels. Darker colors indicate greater variation among all guppy images (N=485) in the color channel measure at a given sample point.

**Appendix Figure S3.** Photography setup for digital image collection. Individual fish were placed within the white lighting tent through the opening visible in panel (A). Panel (B) shows the orientation of the third light source that is not visible in panel (A). The photography setup remained stationary for all sessions within a given year.

**Appendix Figure S4.** Example images of six fish from each of the 11 populations sampled. Panels (A-F) were photographed in 2016, and Panels (G-K) were photographed in 2017. Populations are from the Aripo (A & B), Paria (C), El Cedro (D&E), Marianne (F&G), Guanapo (H&I), and Turure (J&K). Low-predation (LP) populations are pictured to the left of high-predation (HP) populations in each row with the exception of Panel (C) since the Paria River lacks a high-predation contrast.
